# Motor Neuron Susceptibility in ALS/FTD

**DOI:** 10.3389/fnins.2019.00532

**Published:** 2019-06-27

**Authors:** Audrey M. G. Ragagnin, Sina Shadfar, Marta Vidal, Md Shafi Jamali, Julie D. Atkin

**Affiliations:** ^1^Centre for Motor Neuron Disease Research, Department of Biomedical Sciences, Faculty of Medicine and Health Sciences, Macquarie University, Sydney, NSW, Australia; ^2^Department of Biochemistry and Genetics, La Trobe Institute for Molecular Science, La Trobe University, Melbourne, VIC, Australia

**Keywords:** amyotrophic lateral sclerosis, neurodegeneration, selective vulnerability, fast and slow motor units, frontotemporal dementia

## Abstract

Amyotrophic lateral sclerosis (ALS) is a neurodegenerative disease characterized by the death of both upper and lower motor neurons (MNs) in the brain, brainstem and spinal cord. The neurodegenerative mechanisms leading to MN loss in ALS are not fully understood. Importantly, the reasons why MNs are specifically targeted in this disorder are unclear, when the proteins associated genetically or pathologically with ALS are expressed ubiquitously. Furthermore, MNs themselves are not affected equally; specific MNs subpopulations are more susceptible than others in both animal models and human patients. Corticospinal MNs and lower somatic MNs, which innervate voluntary muscles, degenerate more readily than specific subgroups of lower MNs, which remain resistant to degeneration, reflecting the clinical manifestations of ALS. In this review, we discuss the possible factors intrinsic to MNs that render them uniquely susceptible to neurodegeneration in ALS. We also speculate why some MN subpopulations are more vulnerable than others, focusing on both their molecular and physiological properties. Finally, we review the anatomical network and neuronal microenvironment as determinants of MN subtype vulnerability and hence the progression of ALS.

## Introduction

Amyotrophic lateral sclerosis (ALS) is a late-onset, progressive and fatal neurodegenerative disease which primarily affects motor neurons (MNs) of the motor cortex of the brain, brainstem motor nuclei and anterior horn of the spinal cord (Kiernan et al., 2011; Renton et al., 2014; Al Sultan et al., 2016; Taylor et al., 2016). ALS commonly begins in late-adulthood, when patients first experience focal symptoms, such as weakness in the limb or bulbar muscles, as well as widespread fasciculations. The disease then usually progresses in an organized way to adjacent areas of the central nervous system (CNS), and consequently symptoms appear in other regions of the body. Several clinical subsets of ALS can be distinguished by the anatomical location first affected (Renton et al., 2014; Taylor et al., 2016). This includes bulbar onset, where symptoms first appear in the muscles controlling speech, mastication and swallowing; and limb onset, where symptoms present initially in the upper (arm or hand) or lower limbs (leg or foot). Bulbar onset patients face a much worse prognosis than those with spinal onset ALS, where the average survival time following diagnosis is less than 2 years. However, in patients with the much rarer respiratory onset form (3–5%), the prognosis is even worse as the survival time following diagnosis is only 1.4 years (Swinnen and Robberecht, 2014). At disease end stage, only support and palliation are available, and patients usually die from respiratory failure, typically 3–5 years after diagnosis (Taylor et al., 2016). There are currently few effective treatments. Hence there is an urgent need to understand the underlying causes and risk factors for ALS to discover new therapeutic targets.

Neurons have complex and extended morphologies compared to other cell types, and within the CNS, neurons can vary greatly in their properties. MNs are unique cells amongst neurons because they are large, even by neuronal standards, with very long axons, up to 1 m in length in an adult human. MNs can be distinguished into two main categories according to their location in the CNS: upper MNs (UMNs) located in the cortex, and lower MNs (LMNs) located in the brainstem and spinal cord. The spinal MNs comprise both visceral MNs of the thoracic and sacral regions, which control autonomic functions, and somatic MNs, which regulate the contraction of skeletal muscles and thus control movement. The diversity of MNs reflects the variety of targets they innervate, including a wide range of muscle fiber types. UMNs and LMNs differ in the location of their cell bodies, the neurotransmitters released, their targeting and symptoms resulting from their injury.

It is unknown why MNs are specifically targeted in ALS and remarkably, MNs are not equally affected (Rochat et al., 2016; Nijssen et al., 2017). Whilst both UMNs and LMNs are involved, some LMN subtypes are relatively resistant to neurodegeneration. Spinal cord and hypoglossal MNs are amongst the first to degenerate, hence the ability to speak, breath and move is lost early in disease course. As ALS progresses, specific MN subtypes then preferentially deteriorate. However, some MNs are spared until disease end stage, such as oculomotor neurons and Onuf’s nuclei MNs, and as a result, patients retain normal visual, sexual and bladder function throughout the disease course. The resistant MNs differ significantly from the vulnerable MNs anatomically and functionally, and they possess distinct transcriptomes, metabolic and developmental profiles. Surprisingly, there are also differences in vulnerability amongst spinal MNs, because those that are part of the faster motor units degenerate before those in the slower motor units (Frey et al., 2000; Pun et al., 2006; Hegedus et al., 2007; Hadzipasic et al., 2014; Sharma et al., 2016; Spiller et al., 2016a), thus adding further complexity to the question of MN vulnerability.

ALS shares clinical and pathological features with frontotemporal dementia (FTD), a type of dementia that involves impaired judgment and executive skills. In FTD, the loss of cortical MNs is accompanied by loss of neurons in the frontal and temporal cortices, which correlates clinically with the symptoms of FTD (Neumann et al., 2006; Burrell et al., 2016). The relationship between ALS and FTD has been confirmed through genetic studies, and these two conditions are now considered to be at opposite ends of the same disease continuum (Taylor et al., 2016; Shahheydari et al., 2017). Hence, while ALS was historically judged as a disorder affecting the motor system only, it is now recognized that non-motor features are present (Fang et al., 2017). A wealth of evidence also demonstrates that ALS is a heterogeneous disorder. The clinical symptoms, including the proportion of UMN and LMN signs, age of onset, disease duration, and association with other conditions, are major features contributing to its highly variable phenotypes. As well as the development of FTD (Strong and Yang, 2011), ALS can also involve cognitive impairment in up to 50% of patients (Tsermentseli et al., 2012), the autonomic nervous system (Piccione et al., 2015), supranuclear gaze systems (van der Graaff et al., 2009; Donaghy et al., 2011), and extrapyramidal motor signs (Pradat et al., 2002). Sensory, olfactory and visual dysfunction have also been described in some patients (Bede et al., 2016). In addition, there are also other conditions affecting MNs that share similarities, but also striking differences, to ALS. In particular, primary lateral sclerosis (PLS) affects UMNs but it progresses much slowly than ALS. It also has a significantly lower mortality rate (Tartaglia et al., 2007), consistent with the relative resistant of LMNs in ALS.

One of the main pathological characteristics of ALS is the presence of insoluble protein inclusions in the soma of MNs. TAR DNA binding protein-43 (TDP-43) is the major component of these inclusions (Arai et al., 2006; Neumann et al., 2006) in almost all (∼97%) ALS patients and ∼50% FTD patients (Arai et al., 2006; Neumann et al., 2006; Mackenzie et al., 2007; Scotter et al., 2015; Le et al., 2016). Loss of TDP-43 from the nucleus is evident in MNs from ALS/FTD patient tissues, concomitant with the formation of TDP-43 inclusions in the cytoplasm of both MNs and glia. Neuropathological studies have also revealed that the clinical course of ALS reflects the presence of TDP-43 pathology, from its deposition at an initial site of onset, to its spread to contiguous regions of the CNS (Brettschneider et al., 2013). Mutations in TDP-43 are also present in 5% of familial forms of ALS (Sreedharan et al., 2008). In the genetic types of ALS, it remains unclear why MNs are specifically affected when the mutant proteins are ubiquitously expressed. Males are affected more by ALS than females, and ethnic populations show differences in the incidence rates of ALS, further highlighting the contribution of genetics to ALS.

Whilst our understanding of the etiology of ALS has increased significantly in recent years, major gaps in our knowledge remain. In this review, we address several unanswered questions regarding the unique susceptibility of specific types of MNs in ALS: Why does neurodegeneration spread throughout specific neural networks? How can ubiquitously expressed genes be selectively toxic to MNs? Why are some MN subtypes more vulnerable to degeneration than others? We also discuss the role of the neuronal network and the specific cellular microenvironment in driving cell-to-cell disease progression, plus the importance of genetics in influencing susceptibility of specific neuronal subpopulations. Finally, we discuss the role of aging as a potential risk factor for the susceptibility of specific MN subtypes. A thorough comprehension of why specific cell types degenerate is imperative to our understanding of ALS because it provides important clues as to what initiates neurodegeneration, and how this knowledge may be harnessed therapeutically.

## Anatomy of the Motor System

**FIGURE 1:**
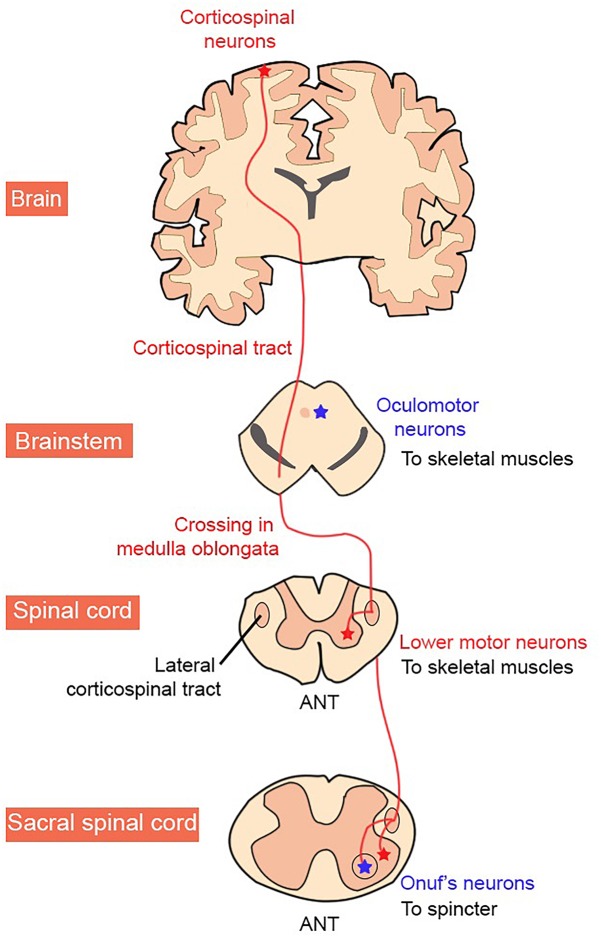
Organization of the human corticospinal tract. MN groups vulnerable and resistant to degeneration in ALS are shown in red and blue, respectively.

In the CNS, the motor cortex, basal ganglia, cerebellum, and parts of the brainstem, are directly involved in the planning and initiation of movement. In contrast, the precise timing and pattern of movement is generated by MNs located in the spinal cord (Figure 1; Kiehn, 2016). The corticospinal (anterior and lateral) tract is the largest descending tract in humans. The lateral corticospinal tract originates in the primary motor cortex, which lies in the precentral gyrus and sends fibers to muscles in the extremity. This is via contralateral cortical innervation, so that the left motor cortex controls the right extremities and vice versa, to control the voluntary movement of contralateral limbs (Javed and Lui, 2018). MNs outputs are not confined to the peripheral muscles however, but also include excitatory terminals to a group of interneurons, Renshaw cells, and also to other MNs.

Glutamate (cortex, spinal cord) and acetylcholine (spinal cord) modulate excitatory input within neurons, whereas GABA and glycine facilitate inhibitory neurotransmission (Ramírez-Jarquín et al., 2014). At the neuromuscular junction (NMJ), only acetylcholine acts at the synapse but interestingly, synaptic transmission between MNs in the spinal cord involves both acetylcholine and glutamate (Bhumbra and Beato, 2018). Renshaw cells are excited through both acetylcholine and glutamate receptors and spinal MNs co-release glutamate to excite Renshaw cells and other MNs, but not to excite muscles (Nishimaru et al., 2005; Bories et al., 2007; Bhumbra and Beato, 2018). Hence, different synaptic transmission systems are present at different postsynaptic targets of MNs (Bhumbra and Beato, 2018).

However, MNs are not homogeneous throughout the CNS because they exhibit distinct morphologies and patterns of connectivity, which underlie their different physiological functions. Hence, within a single region, MNs that perform closely related functions can be further subdivided, both anatomically and physiologically. The identities of specific MN subtypes and their target projections are controlled by selective cell-type expression of transcription factors, notably members of the Hox, LIM, Nkx6, and ETS families (Stifani, 2014). This provides the fundamental mechanism for spinal MN diversification and connectivity to specific peripheral muscle targets. Thus, to generate movement, MNs integrate information from sensory structures and transform it into precise temporal and magnitudal activation of muscles.

A MN located in the spinal cord innervates up to several hundred fibers within one muscle, which together form the motor unit. Trains of action potentials within the axon cause the release of acetylcholine at the NMJ, which activates nicotinic receptors on the muscle fibers the MN innervates. This initiates a cascade of signaling events in the muscle fiber that leads to its contraction. A motor pool consists of all the individual MNs that innervate a single muscle. A muscle unit (one muscle and its motor pool) is composed of three different types of functional motor units consisting of alpha (α), beta (β), and gamma (γ) MNs, which are classified according to the contractile activity of the muscle fiber innervated. We will now discuss in more detail the anatomy of those structures involved in movement.

### The Spinal Cord

In the spinal cord, MNs are organized into columns (Table 1) based on the location of their target muscle [reviewed in Matise and Sharma (2013) and Stifani (2014)]. Within each column, the MNs innervating each muscle are clustered into motor pools, each containing of 20–300 cells depending on the muscle (Bryan et al., 1972; McHanwell and Biscoe, 1981). α-MNs located in the spinal cord are archetypal MNs that innervate extrafusal muscle fibers, thus creating force to move the skeleton (Table 2). In contrast, γ-MNs innervate intrafusal fibers, which modulate the sensitivity of muscle spindles to stretch (Table 2) (Hunt and Kuffler, 1951; Kuffler et al., 1951; Kanning et al., 2010). β-MNs are not as well characterized as α-MNs but they innervate both intrafusal and extrafusal muscle fibers (Bessou et al., 1965). Both α and γ-MNs have large dendritic trees but γ-MNs have fewer large dendrites than α-MNs (7–11) and they also branch less (Westbury, 1982). The somas of γ-MNs are smaller than those of α-MNs and they also possess thinner axons, which reflects their slower conduction velocity (<55 m/s in γ-MN vs. ∼70–90 m/s in α-MNs in cats) (Table 2) (Westbury, 1982). γ-MNs receive only indirect sensory inputs. Therefore, γ-MNs do not directly participate in spinal reflexes (Eccles et al., 1960; Stifani, 2014), but they contribute to the modulation of muscle contraction instead.

**Table 1:** Segmental organization of spinal cord columns.

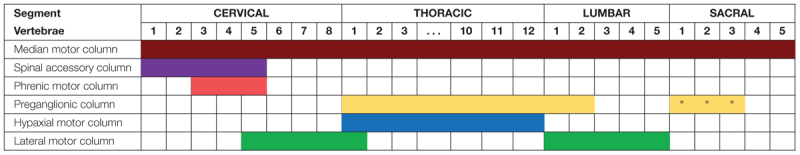

*Pools of MNs that innervate muscles of similar embryonic origin are stereotypically localized within the ventral spinal cord, known as motor columns. The medial motor column (brown) is present in the whole spinal cord and it comprises MNs that innervate the long muscles of the back and the body wall musculature. The spinal accessory column (purple) and the phrenic column (red) are found along the five first cervical segments (C1 to C5) and between C3 and C5, respectively. The preganglionic column (yellow) extends from the first thoracic segment (T1) to the second lumbar segment (L2), and between sacral segments 2 and 4 (S2 to S4) where the Onuf’s nuclei (^∗^) are found. MNs in the preganglionic column innervate neurons of the sympathetic ganglia. The hypaxial motor column (blue) is restricted to the thoracic spinal cord (T1 to T12). The lateral motor column (green), connected to the limbs, comprises the cervical and thoracic spinal cord (from C5 to T1) and the lumbar spinal cord (L1 to L5).*

**Table 2:** Comparison of α- and γ-spinal motor neurons.

	Spinal α-MN	Spinal γ-MN
Target muscle fiber	Extrafusal^1^	Intrafusal^1^
Soma size	Larger^2,3,4,5^	Smaller^2,3,4,5^
Axon diameter	Larger^2^	Thinner^2^
Dendrite branching	More^2^	Less^2^
Motor unit size (innervation ratio)	Larger^6^	Smaller^6^
Membrane input resistance	Larger^7^	Smaller^7^
Firing	Subtype-dependent^8^	Subtype-dependent^8^
Axon conduction velocity	Faster^2,7,9^	Slower^2,7,9^
Afterhyperpolarization duration	Subtype-dependent^7,9^	Variable^7,9^
Spinal reflex	Yes^10^	No^10^
Affected in ALS	Yes^11,12^	Less^11,12^
Affected in aging	Yes^13,14^	No^13,14^
Markers	Osteopontin^15^ RBFOX3/NeuN^16^ Hb9::GFP^5^ NKAα1^17^ (adult)	Err3^16^ Weak NeuN^5,16^ NKAα3^17^ (adult) ESRRG^16^ GFRα1^5^ HTR1D^18^ (early marker) WNT7A^19^ (late embryonic stage)


*ESRRG, estrogen-related receptor gamma; GFRα1, GDNF family receptor alpha 1; HTR1D, serotonin receptor 1D; NAKα1/3, Na^+^/K^+^-ATPases 1/3; RBFOX3, RNA binding protein fox-1 homolog 3. ^*1*^(Kuffler et al., 1951), ^*2*^(Burke et al., 1977), ^*3*^(Westbury, 1982), ^*4*^(Friese et al., 2009), ^*5*^(Shneider et al., 2009), ^*6*^(Adal and Barker, 1965), ^*7*^(Kemm and Westbury, 1978), ^*8*^(Murphy and Martin, 1993), ^*9*^(Gustafsson and Lipski, 1979), ^*10*^(Eccles et al., 1960), ^*11*^(Mohajeri et al., 1998), ^*12*^(Lalancette-Hebert et al., 2016), ^*13*^(Swash and Fox, 1972), ^*14*^(Hashizume et al., 1988), ^*15*^(Misawa et al., 2012), ^*16*^(Friese et al., 2009), ^*17*^(Edwards et al., 2013), ^*18*^(Enjin et al., 2012), ^*19*^(Ashrafi et al., 2012).*

A distinct group of MNs in the sacral spinal cord termed ‘Onuf’s’ neurons, innervate the striated muscles of the external urethra, external anal sphincter via the pudental nerve, and the ischiocavernosus and bulbocavernosus muscles in males (Sato et al., 1978; Nagashima et al., 1979; Kuzuhara et al., 1980; Roppolo et al., 1985). These MNs are histologically similar to limb α-MNs (Mannen et al., 1977) and they are located anteromedial to the anterolateral nucleus and extend between the distal part of the S1 segment and the proximal part of S3.

α-motor units can be subdivided according to their contractile properties, into fast-twitch (F) and slow-twitch (S) fatigue-resistant types (Table 3) (Burke et al., 1973). In addition, fast-twitch α-motor units can be further categorized into fast-twitch fatigable [FF] and fast-twitch fatigue-resistant [FR] types, based on the length of time they sustain contraction. The basis of this classification is the duration of the twitch contraction time (Burke et al., 1973). F- and S-MNs also exhibit different afterhyperpolarization duration (AHP) properties. AHP is the phenomenon by which the membrane potential undershoots the resting potential following an action potential. S-MNs have a longer AHP than F-MNs, indicating that S-MNs have a longer “waiting period” before they can be stimulated by an action potential. Thus, they cannot fire at the same frequency as F-MNs (Eccles et al., 1957), so the larger FF-MNs take longer to reach an activation threshold. Similarly, other electrical properties differ between S- and F-MNs (Table 3), including their input resistance (a measure of resistance over the plasma membrane) and rheobase (a measure of the current needed to generate an action potential). S-MNs have a higher input resistance than F-MNs, underlying Hennenman’s size principle which postulates that S-motor units are the first to be recruited during movement, followed by FR and then FF units (Henneman, 1957; Mendell, 2005). Hence, a slow movement generating a small force will only recruit S-MNs, whereas a quick and strong movement will also recruit F-MNs, as well as S-MNs.

**Table 3:** Comparison of fast (FF, fast-fatigable; FR, fast-resistant) and slow (S) spinal α-motor neurons.

	Spinal α-MN	
	
	F	S
Target muscle fiber	IIb (FF), IIa (FR)^1^	I^1^
Soma size	Similar^2,3,4,5,6^	Similar^2,3,4,5,6^
Axon diameter	Larger^7,8^	Thinner^7,8^
Dendrite branching	More^4,9^	Less^4,9^
Motor unit size (innervation ratio)	Larger^1,10^	Smaller^1,10^
Membrane input resistance	Smaller^11,12,13^	Larger^11,12,13^
Firing	Phasic^14,15^	Tonic^14,15^
Axon conduction velocity	Faster^1,13^	Slower^1,13^
Afterhyperpolarization duration	Shorter^14^	Longer^14^
Recruitment	Late^15^	Early^15^
Affected in ALS	Early^16,17,18^	Late^16,17,18^
Affected in aging	Early^19,20,21^	Late^19,20,21^
Markers	CHODL^22^ CALCA^22^	SV2a^23^ SK3^24^ ESRRB^22^ (adult)


*CALCA, calcitonin-related polypeptide alpha; CHODL, chondrolectin; SV2A, synaptic vesicle glycoprotein 2a; SK3, postsynaptic Ca^2+^-activated K^+^ 3; ESRRB, estrogen-related receptor beta. ^*1*^(Burke et al., 1973), ^*2*^(Kernell and Zwaagstra, 1981), ^*3*^(Burke et al., 1982), ^*4*^(Cullheim et al., 1987), ^*5*^(Vinsant et al., 2013), ^*6*^(Hadzipasic et al., 2014), ^*7*^(Burke et al., 1977), ^*8*^(Dukkipati et al., 2018), ^*9*^(Ulfhake and Kellerth, 1981), ^*10*^(Burke and Tsairis, 1973), ^*11*^(Bakels and Kernell, 1993), ^*12*^(Gardiner, 1993), ^*13*^(Zengel et al., 1985), ^*14*^(Eccles et al., 1957), ^*15*^(Zajac and Faden, 1985), ^*16*^(Frey et al., 2000), ^*17*^(Hegedus et al., 2007), ^*18*^(Pun et al., 2006), ^*19*^(Hashizume et al., 1988), ^*20*^(Kadhiresan et al., 1996), ^*21*^(Kanda and Hashizume, 1989), ^*22*^(Enjin et al., 2010), ^*23*^(Chakkalakal et al., 2010), ^*24*^(Deardorff et al., 2013).*

In addition, at least eleven types of interneurons are involved in the control of movement, as part of central pattern generators in the spinal cord. Interneurons arise from five progenitor cells and, according to the expression of distinct transcription factors, they mature into different lineages. This includes excitatory V2a, V3, MN and Hb9 neurons and inhibitory V0C/G,V0_D_, V0_V_, V1, V2b, Ia and Renshaw cells (belonging to the V1 interneuron subclass), which display specific locations and projections within the spinal cord (Ramírez-Jarquín et al., 2014).

### The Brainstem

Cranial nerve nuclei are populations of neurons in the brainstem that are associated with one or more cranial nerves. They provide afferent and efferent (sensory, motor, and autonomic) innervation to the structures of the head and neck (Sonne and Lopez-Ojeda, 2018). The more posterior and lateral nuclei tend to be sensory, and the more anterior nuclei are usually motor nuclei. Trigeminal MNs innervate the muscles of mastication, whereas facial MNs supply the superficial muscles of the face, and ambiguous MNs supply the muscles of the soft palate, pharynx, and larynx. The oculomotor (III), trochlear (IV) and abducens (VI) nuclei are somatic efferents innervating the extraocular muscles within the orbit. The oculomotor nucleus contains MNs that innervate four of the six extraocular muscles (superior, medial and inferior recti, inferior oblique), plus the levator palpebrae superioris muscle. These muscles display a unique composition of six fiber types, distinct from other skeletal muscles that possess marked fatigue resistance (Table 4). Oculomotor units are amongst the smallest of the motor units, in contrast to skeletal muscle motor units that have higher maximum MN discharge rates. Furthermore, α-MNs in oculomotor units have higher resting membrane potentials (∼61 mV) than spinal cord α-MNs (∼70 mV), and they also discharge at higher frequencies (∼100 Hz during steady state and ∼600 Hz during saccadic eye movements, compared to ∼100 Hz for spinal cord α-MNs) (Table 4) (Robinson, 1970; Fuchs et al., 1988; Torres-Torrelo et al., 2012). Oculomotor neurons are almost continually active at high frequencies when maintaining eye position (Fuchs et al., 1988; De La Cruz et al., 1989), and this level of activity places high metabolic demand on these cells (Robinson, 1970; Porter and Baker, 1996; Brockington et al., 2013).

**Table 4:** Comparison of α-spinal motor neurons and oculomotor neurons.

	Spinal α-MN	Oculomotor neuron
Target muscle fiber	Single fiber type^1^	Multiple fiber types^1^
Soma size	Larger^2,3^	Smaller^2,3^
Dendrite branching	Larger^2^	Smaller^2^
Motor unit size (innervation ratio)	Larger^4,5,6,7^	Smaller^4,5,6,7^
Resting potential	Smaller^8,9,10^	Higher^8,9,10^
Discharge frequency	100 Hz^8,9,10^	100–600 Hz^8,9,10^
Affected in ALS	Yes^11,12,13,14^	No^11,12,13,14^
Affected in aging	Yes^15,16^	No^15,16^


*^*1*^(Zhou et al., 2011), ^*2*^(Durand, 1989), ^*3*^(Shoenfeld et al., 2014), ^*4*^(Burke et al., 1971), ^*5*^(Burke and Tsairis, 1973), ^*6*^(Enoka, 1995), ^*7*^(Guéritaud et al., 1985), ^*8*^(Robinson, 1970), ^*9*^(Fuchs et al., 1988), ^*10*^(Torres-Torrelo et al., 2012), ^*11*^(Nimchinsky et al., 2000), ^*12*^(Hedlund et al., 2010), ^*13*^(Valdez et al., 2012), ^*14*^(Comley et al., 2016), ^*15*^(Hashizume et al., 1988), ^*16*^(Swash and Fox, 1972).*

### The Cortical Motor System

The motor cortex is the region of the cerebral cortex responsible for mediating voluntary movements. In rodents, the primary cortex (M1) is large and comprises almost all of the frontal cortex (Gioanni and Lamarche, 1985; Neafsey et al., 1986; Brecht et al., 2004; Yu et al., 2008; Hira et al., 2013; Paxinos, 2014), whereas in primates, the frontal cortex is compartmentalized into specialized premotor subfields and M1 is relatively small in comparison (Ferrier, 1875; Leyton and Sherrington, 1917; Asanuma and Rosén, 1972; Dickey et al., 2013; Riehle et al., 2013; Young et al., 2013; Ebbesen and Brecht, 2017). M1 plays a central role in controlling movement. This involves specialized UMNs located in layer V of this region (Broadman area 4), the giant Betz cells or corticospinal MNs. These MNs are the cortical components of the MN circuit that initiates and modulates precise voluntary movement, through long-range projections to the spinal cord. Approximately ∼30–50% of corticospinal projections originate from M1 MNs and they begin modulating their firing rate several hundred ms before movement of the limb is initiated (Georgopoulos et al., 1982; Porter and Lemon, 1993). In most mammals, the axons of cortical MNs terminate at spinal interneurons, but they also make direct connections to MNs (Lemon, 2008; Rathelot and Strick, 2009). This constitutes the final efferent pathway to the muscle to generate or suppress movement (Ramírez-Jarquín and Tapia, 2018).

### Motor Neurons Selectively Degenerate in ALS Patients

Lesions to motor structures in humans and experimental animals lead to impairments in normal movement. In ALS, as MNs degenerate, the ability to control movement of the muscles is progressively lost. Specific MNs in the brain, brainstem and spinal cord are selectively targeted, and pathology appears first in these restricted MN populations. In fact, the name “Amyotrophic Lateral Sclerosis” reflects the strikingly selective degeneration of MNs in ALS. It is derived from a combination of three words; “Lateral” refers to the lateral spinal cord, given that corticospinal MNs are particularly vulnerable to degeneration; “Amyotrophic” is from the Greek “amyotrophia,” meaning lacking muscle nourishment; and “Sclerosis” (fibrosis) refers to gliosis of the crossed corticospinal tract in the dorsolateral quadrant of the spinal cord (Charcot, 1874; Frey et al., 2000; Pun et al., 2006). In the brain, UMNs in the primary cortex are also amongst the first to degenerate in ALS, and similarly, in the brainstem, the hypoglossal MNs that innervate the muscles of the tongue involved in swallowing and breathing, are also targeted early in disease course. In the brainstem, ALS can also affect trigeminal MNs, the facial MNs and ambiguous MNs. However, other MN subgroups within this region are relatively resistant to degeneration, including MNs of the oculomotor (III), trochlear (IV) and abducens (VI) nuclei, innervating the extraocular muscles (Mannen et al., 1977; Schrøder and Reske-Nielsen, 1984). Hence, eye movements remain relatively preserved throughout disease course (Kanning et al., 2010) and as a consequence, eye tracking devices are often used to aid communication in the later stages of ALS (Caligari et al., 2013). Whilst it has been reported that oculomotor neurons may be affected at disease end stage, this was recently attributed to dysfunction of the dorsolateral prefrontal cortex, the frontal eye field and the supplementary eye field, confirming the relative resistance of pure oculomotor functions in ALS (Shaunak et al., 1995; Proudfoot et al., 2015). Widespread loss of GABAergic interneurons has also been described in ALS, in both the cortex (Stephens et al., 2001; Maekawa et al., 2004) and the spinal cord (Stephens et al., 2006; Hossaini et al., 2011).

MRI studies of ALS patients has revealed that very specific neuronal networks are vulnerable to degeneration in ALS (Bede et al., 2016). However, whilst TDP-43 pathology is the signature pathological hallmark of almost all ALS cases, it can arise in areas of the CNS that are not particularly vulnerable to degeneration (Geser et al., 2008). Significant TDP-43 pathology is present in the substantia nigra and basal ganglia, which are not affected in ALS, as well as in the motor gyrus, midbrain and spinal cord. Curiously, pathological forms of TDP-43 are also detectable in the occipital lobe, amygdala, orbital gyrus and hippocampus (Geser et al., 2008). Hence, whilst major degeneration of corticobulbar, LMN, pyramidal and frontotemporal networks underlie the widespread clinical symptoms of ALS, it remains unclear how other circuits, such as the visual, sensory, autonomic and auditory systems, remain relatively protected in ALS. These unaffected networks, however, have not been well studied in ALS patients.

## Genetic Mutations and Risk Factors in ALS

### Genetics of ALS

**FIGURE 2:**
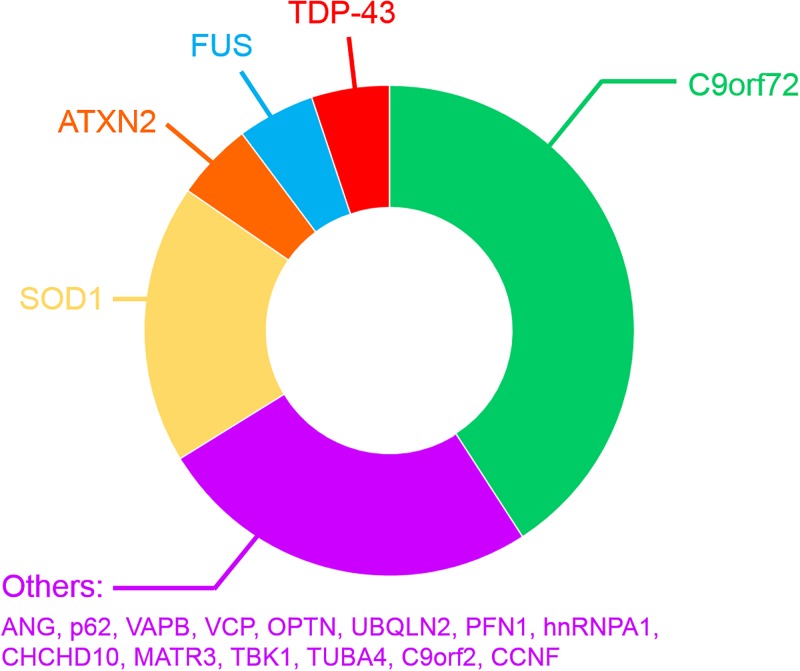
Frequency of mutated genes in FALS patients.

Most ALS cases occur without a clearly identified cause and are therefore referred to as sporadic ALS (SALS). In contrast, a positive family history is present in ∼10% of all patients (familial ALS; FALS) (van Blitterswijk et al., 2012; Nguyen et al., 2018) and these genetic mutations cause ALS in a mostly autosomal-dominant manner (Supplementary Table 1 and Figure 2). However, several recently discovered mutations have been described in patients diagnosed with SALS (Renton et al., 2014; Al Sultan et al., 2016; Taylor et al., 2016). The patterns of selective MN degeneration and vulnerability are similar between FALS and SALS (Comley et al., 2015), implying that shared molecular mechanisms exist between the two conditions.

The first gene found to harbor mutations causing FALS encodes Cu/Zn superoxide dismutase (*SOD1*), an enzyme that detoxifies superoxide radicals (Rosen et al., 1993). Mutations in *SOD1* account for 12–23.5% of FALS cases, representing 1–2.5% of all ALS, and 186 ALS mutations have now been described^1^. Since then, mutations in approximatively 26 genes have been identified (Supplementary Table 1 and Figure 2) using genome-wide or exome-wide association studies combined with segregation analysis. Hexanucleotide repeat expansions (GGGGCC) within the first intron of the chromosome 9 open reading frame 72 (*C9orf72*) gene are the most common cause of FALS and FTD (∼30–50% of FALS, ∼10% of SALS 25% of familial FTD and ∼5% of apparently sporadic ALS and FTD) (DeJesus-Hernandez et al., 2011b; Renton et al., 2011; Majounie et al., 2012; Devenney et al., 2014) (Supplementary Table 1 and Figure 2), in both Europe and North America (DeJesus-Hernandez et al., 2011b; Renton et al., 2011). However, this mutation is much rarer in Asian and Middle Eastern populations (Majounie et al., 2012; Woollacott and Mead, 2014). Healthy individuals possess ≤ 11 GGGGCC repeats in *C9orf72* (Rutherford et al., 2012; Harms et al., 2013; van der Zee et al., 2013), whereas hundreds to thousands of repeats are present in ALS/FTD patients (Beck et al., 2013; Harms et al., 2013; van Blitterswijk et al., 2013; Suh et al., 2015). After *C9orf7*2, mutations in *SOD1* (20% of FALS), *TARDPB* encoding TDP-43 (5% of FALS, >50% of FTD) (Rutherford et al., 2008; Sreedharan et al., 2008; Borroni et al., 2010; Kirby et al., 2010), *Fused in sarcoma* encoding FUS (*FUS*, 5% of FALS) (Belzil et al., 2009; Blair et al., 2009; Chiò et al., 2009; Kwiatkowski et al., 2009; Neumann et al., 2009; Vance et al., 2009), and *CCNF* encoding cyclin F (0.6–3.3% of FALS-FTD) are more frequent than the remaining 20 genes mutated in the much rarer forms of FALS (Supplementary Table 1). The physiological functions and properties of the proteins encoded by these genes can be grouped according to their involvement in protein quality control, cytoskeletal dynamics, RNA homeostasis and the DNA damage response. However, it is possible that genetic inheritance could sometimes be missed, due to incomplete penetrance or an oligogenic mode of inheritance, whereby more than one mutated gene is necessary to fully present disease (Nguyen et al., 2018). Consistent with this notion, the frequency of ALS patients carrying two or more mutations in ALS-associated genes is in excess of what would be expected by chance (van Blitterswijk et al., 2012; Veldink, 2017; Zou et al., 2017; Nguyen et al., 2018).

TDP-43 is an ubiquitously expressed RNA-binding protein belonging to the heterogeneous nuclear ribonucleoprotein (hnRNP) family. Fifty three mutations in *TARDBP* have now been associated with FALS, located within all but one reside of the C-terminal domain of TDP-43 [Gitcho et al., 2008; Kabashi et al., 2008; Van Deerlin et al., 2008; http://alsod.iop.kcl.ac.uk/]. Pathological forms of TDP-43 – phosphorylated, fragmented, aggregated, ubiquitinated TDP-43 – were identified as the major component of MN inclusions (Neumann et al., 2006) in almost all ALS cases, including SALS (97%) (Arai et al., 2006; Neumann et al., 2006; Mackenzie et al., 2007; Scotter et al., 2015; Le et al., 2016). TDP-43 pathology is also observed in *C9orf72* mutation cases in several brain regions, including the frontal, temporal and primary motor cortices, hippocampus, basal ganglia, amygdala, thalamus and midbrain (Murray et al., 2011; Hsiung et al., 2012; Mahoney et al., 2012; Irwin et al., 2013; Mackenzie et al., 2013; Balendra and Isaacs, 2018), highlighting an important role for TDP-43 in neurodegeneration in both SALS and FALS. Moreover, ALS and FTD cases bearing TDP-43 pathology are often referred to “TDP-43 proteinopathies” (Mackenzie et al., 2009). TDP-43 shares similar functional roles in RNA-binding, splicing and nucleocytosolic RNA transport as FUS. Fifty nine mutations in FUS have been identified in both SALS and FALS patients (Lattante et al., 2013; http://alsod.iop.kcl.ac.uk/) and FUS colocalises with TDP-43 in protein aggregates in MNs of a proportion of SALS and FALS patients (Kwiatkowski et al., 2009; Deng et al., 2010).

### Disease Mechanisms Implicated in ALS

A wide range of cellular pathways have been implicated in ALS pathogenesis, as reviewed recently (Shin and Lee, 2013; Taylor et al., 2016; Balendra and Isaacs, 2018). These include altered RNA processing/metabolism, nucleolar dysfunction, RNA splicing transcriptional defects (Barmada, 2015; Fratta and Isaacs, 2018) and DNA damage (Konopka and Atkin, 2018; Penndorf et al., 2018). Proteostasis pathways have also been implicated, with impairments in autophagy and lysosomal function, the endoplasmic reticulum (ER), mitochondrial and the ubiquitin–proteasome systems described (Maharjan and Saxena, 2016; Ruegsegger and Saxena, 2016). Furthermore, several modes of vesicular trafficking are impaired in ALS, including nucleocytoplasmic (Kim and Taylor, 2017), ER-Golgi (Soo et al., 2015), and axonal forms of transport (De Vos and Hafezparast, 2017). In addition, defects in neuronal-specific processes, including hyper-excitability and hypo-excitability, glutamate excitotoxicity, and neuronal branching defects, have also been described in ALS (Fogarty, 2018).

### Mouse Models of ALS

Over the last 20 years, several transgenic mouse strains expressing human mutant SOD1 have been generated. These mice have been used to either examine disease mechanisms or trial potential therapeutic strategies for ALS, although the latter has led to questionable success (Perrin, 2014) (Tables 5, 6). The transgenic line harboring the Gly93 → Ala substitution (SOD1^G93A^) has been used most extensively (Gurney et al., 1994), followed by the SOD1^G37R^ (Wong et al., 1995), SOD1^G85R^ (Bruijn et al., 1997), SOD1^G86R^ (Ripps et al., 1995) and SOD1^D90A^ (Jonsson et al., 2006) models.

**Table 5:** SOD1, TDP-43 and FUS mouse models of ALS.

Mouse models		Promotor	CNS over-expression (fold)	Survival (months)	Inclusions	Motor Phenotype	MN loss	Denervation	References
SOD1	G93A	hSOD1	17	3.5–4.5	SOD1(+)	Yes	Yes	Yes	Gurney et al., 1994
	s-G93A	hSOD1	8–10	8.3	hyaline	Yes	Yes	Yes	Gurney, 1997
	G37R	hSOD1	4–12	5	SOD1(+)	Yes	Yes	Yes	Wong et al., 1995
	G85R	hSOD1	0.2–1	8.5	SOD1(+) Ub(+)	Yes	Yes	Yes	Bruijn et al., 1997
TDP-43	A315T	*PrP*	3	5	TDP-43(–) Ub(+)	Yes	Yes	Yes	Wegorzewska et al., 2009
	rNLS8	*NEFH*	–	2.6 off Dox	TDP-43(+)	Yes	Yes	Yes	Walker et al., 2015; Spiller et al., 2016a
	M337V^KNOCK-IN^	–	No	24.5	No	No	No	Yes	Ebstein et al., 2019
	G298S^KNOCK-IN^	–	No	24.5	No	No	Yes	Yes	Ebstein et al., 2019
	TDP-43 KO	–	–	ns	No	Yes	Yes	Yes	Iguchi et al., 2013
FUS	hFUS^WT^	*MAPT*		2.6	No	No	No	Yes	Sharma et al., 2016
	hFUS^R521C^	*MAPT*	4	12	No	No	Yes	Yes	Sharma et al., 2016
	hFUS^P525L^	*MAPT*	4	12	No	Yes	Yes	Yes	Sharma et al., 2016


**Table 6:** Commonly used SOD1-transgenic mouse models of ALS and their phenotypes in relation to transgenic expression.

SOD1 mouse models	Transgene copies	SOD1 protein levels in the CNS (human/mouse)	Disease onset (days)	Survival (months)	References
B6SJL-TgN(SOD1-G93A)1Gur	34	17	90	3.5–4.5	Gurney et al., 1994; Alexander et al., 2004
SOD1-G93A Drop Copy#3	13	–	–	6	Alexander et al., 2004
SOD1-G93A Drop Copy#4	11	–	–	6.5	Alexander et al., 2004
B6SJL-TgN(SOD1-G93A)^dl^1Gur	10	8–10	168	8.3	Gurney, 1997
SOD1-G93A Drop Copy#1	4	–	–	21	Alexander et al., 2004
G37R	–	4–12	105	5	Wong et al., 1995; Haenggeli et al., 2007
G85R	–	0.2–1	240	8.5	Bruijn et al., 1997
G86R (M1 line)	–	–	90–120	4	Ripps et al., 1995
D90A	–	–	350	13.5	Jonsson et al., 2006


*(–), unknown*.

The B6SJL-TgN(SOD1-G93A)1Gur mouse (Gurney et al., 1994) carries 25 ± 1.5 copies of the transgene within chromosome 12 and as a result, it expresses very high levels of human mutant SOD1^G93A^ (Alexander et al., 2004). Whilst these significant levels of overexpression are criticized as a major limitation (Alexander et al., 2004), these animals remain the most widely used mouse model for therapeutic studies in ALS (Gurney et al., 1994). These SOD1^G93A^ mice become paralyzed in the hindlimbs as a result of MN loss from the spinal cord, resulting in death by 5 months of age. Another variant of this model, B6SJL-TgN(SOD1-G93A)^dl^1Gur, possesses fewer copies of the transgene; 8 ± 1.5 (Gurney, 1997; Alexander et al., 2004)^2^. This “low-copy” mouse, hereafter referred to as “G93A-slow” (s-SOD1^G93A^), develops a slower disease course in comparison, where paralysis begins at 6–8.5 months of age (Alexander et al., 2004; Muller et al., 2008; Acevedo-Arozena et al., 2011). In addition, several other “low-copy” mouse lines have subsequently been generated, with even fewer copies of the human SOD1^G93A^ transgene. These models also exhibit greater life spans compared to the higher copy lines (Alexander et al., 2004) (Table 6). Similarly, four lines of mice expressing another SOD1 mutant, SOD1^G37R^, at different levels (5–14 times) have been produced, with variable phenotypes (Wong et al., 1995). Multiple mouse models based on transgenic expression of wild type or mutant TDP-43 have also been generated (Philips and Rothstein, 2015) (Table 5). Overexpressing human TDP-43 with a defective nuclear localization signal (NLS) in mice – in the absence of an ALS mutation – results in cytoplasmic expression of hTDP-43 and nuclear TDP-43 clearance. This results in a severe motor phenotype and reduced survival in the resulting ‘rNLS8’ mice compared to littermate controls (Walker et al., 2015). Several mouse models also exist based on transgenic expression of mutant FUS (Table 5). These mice display progressive, age- and mutation-dependent degeneration that also model aspects of ALS (Sharma et al., 2016). Furthermore, several newer models based on the C9orf72 repeat expansion have also been produced, although the phenotypes are more reminiscent of FTD rather than ALS (Batra and Lee, 2017).

### Misfolded Protein Expression Level Influences Susceptibility

The expression of specific proteins can vary between MN subpopulations and this may be linked to their vulnerability to degenerate. Evidence for this hypothesis comes from the existing mouse models of ALS. Whilst mutant SOD1^G93A^ is expressed in all MNs in these mice (Jaarsma et al., 2008), its propensity to induce neurodegeneration and disease is proportional to its expression level (Table 6) (Gurney et al., 1994; Bruijn et al., 1997; Alexander et al., 2004). At lower levels of expression, pathology is restricted to MNs in the spinal cord and brainstem only, whereas higher expression levels also induce severe abnormalities in the brain. Fewer copies of the SOD1^G37R^ transgene correlate with delayed disease progression and a significant increase in lifespan compared to animals with higher copy numbers (Table 6) (Zwiegers et al., 2014). Similarly, in TDP-43 models, higher levels of overexpression are associated with a worse phenotype (Philips and Rothstein, 2015). Moreover, disease is evident in both wildtype and mutant TDP-43 models, indicating that the expression levels of TDP-43, rather than the presence of a mutation *per se*, induces neurodegeneration. Hence, the effect of the TDP-43 mutation can be difficult to segregate from the effects of overexpression in these models (Philips and Rothstein, 2015). Both retaining the physiological expression levels and normal nuclear localization of TDP-43 have been linked to maintaining cellular homeostasis (Swarup et al., 2011; Philips and Rothstein, 2015). These studies together highlight the role of differing protein expression levels in the development and progression of ALS. However, further work is required to determine whether the expression levels of mutant ALS-associated proteins are different among MN subtypes, and whether this can differentially sensitize specific MNs to neurodegeneration and stress in ALS.

### Selectivity in MN Degeneration in Mouse Models of ALS

Rodent disease models are also useful in studies examining the selective vulnerability of specific MNs within an individual motor pool in ALS. Similar to human ALS, in mouse models based on mutant SOD1^G93A^, TDP-43^A315T^ and FUS^P525L^, α-MNs selectively degenerate, while γ-MNs and MNs in the Onuf’s nucleus are spared (Mannen et al., 1977; Lalancette-Hebert et al., 2016). Also, as in ALS patients, the oculomotor MNs are spared in SOD1^G93A^ (Niessen et al., 2006) and SOD1^G86R^ (Nimchinsky et al., 2000) mice, whereas spinal cord MNs, trigeminal, facial and hypoglossal MNs are targeted (Niessen et al., 2006). In rNLS8 mice, MNs in the hypoglossal nucleus and the spinal cord are also involved, whereas those in the oculomotor, trigeminal, and facial nuclei are spared, despite widespread neuronal expression of cytoplasmic hTDP-43 (Spiller et al., 2016a). Atrophy of MNs in the trigeminal motor, facial and hypoglossal nuclei are also significantly smaller in TDP-43 knock-out mice, whereas MNs in the oculomotor nuclei are preserved (Iguchi et al., 2013). In addition, in another TDP-43 model, Prp-TDP43^A315T^ mice, degeneration of specific neuronal populations occurs (Wegorzewska et al., 2009). Cytoplasmic ubiquitinated proteins accumulate in neurons of cortical layer V and in large neurons of the ventral horn and scattered interneurons, despite expression of the Prp-TDP-43^A315T^ transgene in all neurons and glia (Wegorzewska et al., 2009). In a knock-in TDP-43 mouse model bearing a G298S mutation, MN loss was restricted to large-diameter α-MNs (Ebstein et al., 2019). Furthermore, in FUS^P525L^ and FUS^R521C^ mouse models, no significant MN loss was detected in oculomotor neurons, whereas spinal cord MNs were progressively lost during disease course (Sharma et al., 2016).

In mutant SOD1^G93A^ mice, FF α-MNs are more susceptible to degenerate than FR α-MNs, resulting in the FF muscles becoming paralyzed before FR muscles (Hegedus et al., 2007). Furthermore, tonic S-units only disconnect from the muscle at disease end stage, meaning that S α-MNs are the least vulnerable within motor pools in SOD1^G93A^, SOD1^G85R^ (Frey et al., 2000; Pun et al., 2006; Hegedus et al., 2007; Hadzipasic et al., 2014), TDP-43 rNLS8 (Spiller et al., 2016a), FUS^R521C^ and FUS^P525L^ transgenic models (Sharma et al., 2016). These findings together therefore provide strong evidence that there is a gradient of vulnerability amongst spinal MNs, whereby the faster, less excitable motor units are affected before the slower, more excitable types, at least in mouse models. Interestingly, selective denervation of MN subtypes occurs at the NMJ. Less denervation of the relatively resistant slow-twitch soleus muscle (Frey et al., 2000), compared to the vulnerable fast-twitch tibialis anterior muscle, occurs in TDP-43^M337V^, TDP-43^G298S^, FUS^P525L^, FUS^R521C^ and TDP-43 rNLS8 mouse models (Sharma et al., 2016; Spiller et al., 2016a; Ebstein et al., 2019). In both the low- and high-copy s-SOD1^G93A^ and SOD1^G93A^ mice, the onset of interneuron degeneration also precedes the onset of behavioral motor manifestations and most MN degeneration (Chang and Martin, 2009; Jiang et al., 2009; Pullen and Athanasiou, 2009). Subtle changes to inhibitory synaptic inputs to MNs may therefore modulate MN excitability, leading to degeneration and motor symptoms in ALS/FTD.

## Network-Driven MN Vulnerability

Genetic mutations are present throughout life in ALS patients (summarized in Supplementary Table 1), but as only specific cellular populations are affected, this implies that the vulnerability of MN subtypes in ALS is not caused wholly by genetic factors. Hence, environmental or extrinsic factors, such as the neuronal circuitry or the microenvironment surrounding MNs, may explain the selective vulnerability of MNs in ALS/FTD.

### Site-Specific Onset and Spread of Neurodegeneration in ALS

The pattern of neurodegeneration in ALS/FTD is not random; it targets specific large-scale distributed networks in the brain and spinal cord. Motor manifestations begin in one region of the body in ∼98% of patients (Ravits et al., 2007) accompanied by unilateral, focal damage to MNs in the motor cortex or spinal cord, that innervate the corresponding peripheral body regions. It has been previously suggested that ALS targets specific evolutionarily linked, interdependent functions, and as the disease progresses these deficits combine into failure of specific networks (Eisen et al., 2014). More recently, several clinical studies have revealed that neurodegeneration and TDP-43 pathology spread to continuous anatomical regions during disease course (Ravits et al., 2007; Brettschneider et al., 2013; Walhout et al., 2018), and symptoms arise in the contralateral regions following a unilateral limb onset (Walhout et al., 2018). This also implies that neuronal circuitry might drive disease progression to specific MN populations in ALS/FTD. The spread of misfolded proteins from cell-to-cell, particularly TDP-43, provides a molecular explanation for the specific network and anatomical vulnerability observed in ALS. However, it must be noted that whilst contiguous spread is observed for most patients, this is not the case for all (Ravits and La Spada, 2009).

Increasing evidence suggests that ALS begins in the cortical regions of the brain, which is referred to as the “dying-forward hypothesis.” Features of cortical hyperexcitability – heralded by reduction in short interval intracortical inhibition – have been detected during the early phases of ALS in transcranial magnetic stimulation studies (Thomsen et al., 2014; Menon et al., 2015). This can precede the clinical onset of bulbar/spinal motor dysfunction by ∼3–6 months (Vucic et al., 2008; Bakulin et al., 2016). The dying forward hypothesis is consistent with Charcot, who first postulated that ALS begins in the cortex (Charcot, 1874). Clinical observations that MNs without monosynaptic connections to cortical MNs, such as the oculomotor, abducens, and Onuf’s nuclei, are spared in ALS, and that pure LMN forms of ALS are rare, also support this hypothesis. Further evidence is provided by the observation that MNs receiving direct, monosynaptic cortical input also predominantly develop TDP-43 pathology, while subcortical MNs do not (Eisen et al., 2017). Similarly, TDP-43 pathology develops in patients only in structures under the control of corticofugal projections (Brettschneider et al., 2013; Menon et al., 2015; Eisen et al., 2017)

**FIGURE 3:**
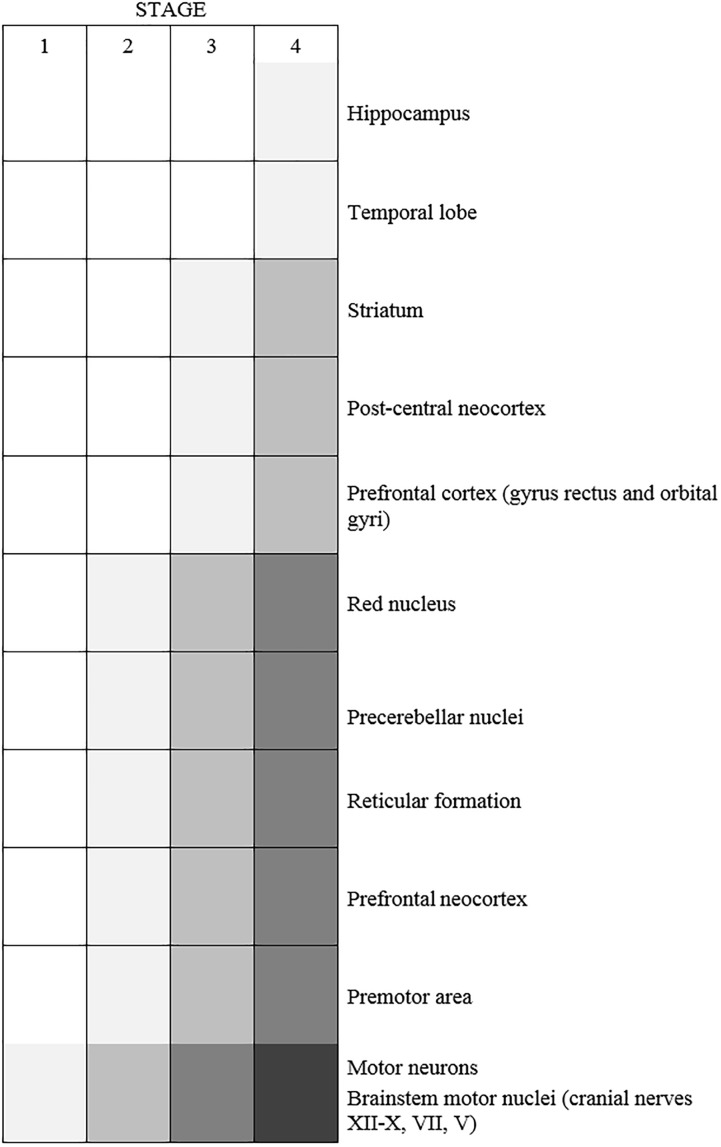
Schematic diagram representing the typical spread of neurodegeneration following an initial onset in motor neurons in ALS patients (*n* = 76 patients) (Brettschneider et al., 2013). Shading represents TDP-43 pathology.

TDP-43 pathology may then propagate through corticofugal axons to the spinal cord and regions of the brain (Braak et al., 2013; Eisen et al., 2017) in a time-dependant and region-specific manner (Brettschneider et al., 2013), consistent with the dying forward hypothesis (Figure 3). This sequential pattern of TDP-43 dissemination is consistent with the hypothesis that TDP-43 pathology is propagated synaptically from cell to cell (Brundin et al., 2010; Maniecka and Polymenidou, 2015), in a similar way to the pathogenic prion protein, a concept known as the “prion-like mechanism” (Lee and Kim, 2015; Ayers and Cashman, 2018). In this model, misfolded proteins act as template seeds to trigger aggregation of their natively folded counterparts. This results in the propagation of protein misfolding, leading to its orderly spread through the CNS (Soto, 2012; Maniecka and Polymenidou, 2015). However, the question of where disease begins remains controversial because many researchers still favor the “dying-back” hypothesis, in which ALS begins within the muscle cells or at the NMJ. This hypothesis proposes that there is a spread of pathology from LMNs to UMNs (Chou and Norris, 1993; Fischer et al., 2004; Pun et al., 2006; Turner et al., 2018), or else, a simultaneous involvement of both UMNS and LMNs (Turner et al., 2018). Whilst most of the evidence for the dying-back mechanism comes from animal models, studies of muscle biopsies from early stage ALS patients and long-term survivors have demonstrated significant morphological abnormalities and major denervation/re-innervation at the NMJ, implying that this region is targeted early in disease (Millecamps et al., 2010; reviewed in Arbour et al., 2017).

There is evidence to support the prion-like model in ALS. The spread of neurodegeneration through adjacent anatomical regions of the CNS resembles the orderly spread of protein misfolding in prion disease. The *in vitro* cell-to-cell transmission of misfolded SOD1, TDP-43 and C9orf72 di-peptide repeat proteins has been demonstrated (Grad et al., 2011, 2014; Münch et al., 2011; Nonaka et al., 2013; Feiler et al., 2015; Porta et al., 2018). Similarly, the addition of cerebrospinal fluid from ALS/FTD patients (Ding et al., 2015), detergent-insoluble fractions of ALS-disease brains (Nonaka et al., 2013) or insoluble phosphorylated TDP-43 from post-mortem brain and spinal cord tissue (Smethurst et al., 2016), results in misfolding of TDP-43 when added to human cell lines. However, so far, only misfolded SOD1 and TDP-43 transmissibility has been demonstrated *in vivo* (Ayers et al., 2014, 2016; Porta et al., 2018). A recent study demonstrated that injection of brain-derived extracts from FTD patients into mice promoted the spatio-temporal transmission of TDP-43 pathology via the neuroanatomical connectome, suggesting that TDP-43 travels via axonal transport through connected regions of the CNS (Porta et al., 2018). Similarly, axonal transport is implicated in the spread of mutant SOD1 in mice (Ayers et al., 2016). Overexpression of misfolded TDP-43 or SOD1 facilitated the seeding ability of each inoculum, consistent with results obtained *in vitro* (Nonaka et al., 2013; Feiler et al., 2015; Smethurst et al., 2016).

Whilst these animal studies demonstrate that ALS spreads within MNs that are connected synaptically, a small portion of patients do not display this contiguous spreading of pathology, however. This implies the existence of alternative mechanisms of disease progression (Fujimura-Kiyono et al., 2011; Gargiulo-Monachelli et al., 2012), such as the transfer of misfolded proteins in nanotubules or exosomes (Nonaka et al., 2013; Sundaramoorthy et al., 2013; Grad et al., 2014; Ding et al., 2015; Feiler et al., 2015; Westergard et al., 2016). Interestingly, it has been suggested that the vulnerability of specific MN populations is associated with the spread of neurodegeneration in ALS (Fu et al., 2018).

### Role of Glial Cells in Driving Disease Progression

There is increasing evidence for a role of the neighboring non-neuronal cells in ALS. Under normal conditions, glial cells provide nutritional and trophic support to MNs, but in ALS, they appear to exacerbate neurodegeneration in a non-cell autonomous fashion. These cells include microglia, astrocytes, oligodendrocytes and Schwann cells. Limiting the expression of mutant SOD1 to MNs only does not lead to neurodegeneration in mice (Pramatarova et al., 2001; Lino et al., 2002), and chimeric mouse studies have established that the presence of mutant SOD1^G93A^ in glial cells induces neurodegeneration and MN loss (Papadeas et al., 2011). Both microglia and astrocytes appear to enhance disease progression by inducing neuroinflammation, whereas oligodendrocytes drive disease initiation. Non-neuronal cells may also be involved in the spread of pathological proteins in ALS (Thomas et al., 2017; Porta et al., 2018). However, whilst misfolded proteins released by MNs can be taken up by glial cells, they may be less toxic to these cells than to MNs (Benkler et al., 2018).

#### Microglia

Microglia are the main immune cells of the CNS (Fujita and Kitamura, 1975; Hickey and Kimura, 1988; Lawson et al., 1990). In ALS patients, activated microglia increase in CNS regions that are susceptible to neurodegeneration (Kawamata et al., 1992) and in SOD1^G93A^ mice, enhanced microglial reactivity precedes nerve denervation at the NMJ (Alexianu et al., 2001; Saxena et al., 2009). Microglia exist in both resting and activated states [reviewed in Perry and Holmes (2014)] and in ALS, activated microglia display two distinct phenotypes. The neuroprotective M2 phenotype promotes tissue repair and supports MN survival by releasing neuroprotective factors, and the toxic M1 phenotype produces cytokines, enhances inflammation, and induces cell death (Liao et al., 2012). Studies in mutant SOD1 mice reveal that the numbers of microglia increase during disease progression, but they vary between the neuroprotective M2 and toxic M1 phenotypes (Liao et al., 2012; Chiu et al., 2013). In lumbar spinal cords of pre-symptomatic SOD1^G93A^ mice, the anti-inflammatory M2 microglia predominate (Gravel et al., 2016), whereas at disease onset and during progression, the proinflammatory M1 type is more common (Beers et al., 2011). Microglial-specific ablation of mutant SOD1^G37R^ in mice does not affect disease initiation, but it significantly slows disease progression (Boillée et al., 2006b), indicating that microglia enhance the progression, but not the onset, of disease in transgenic mutant SOD1 mice. However, contradictory findings were obtained in the TDP-43 rNLS8 model, where microglia were neuroprotective and not neurotoxic (Spiller et al., 2018). Interestingly, knockdown of C9orf72 in mice alters microglial function and induces age-related neuroinflammation, but not neurodegeneration (Lall and Baloh, 2017). Further investigations are required to examine the role of microglia in other ALS disease models, and to determine whether MN subtypes display different vulnerabilities to microglia-mediated protective and/or toxicity in ALS.

#### Astrocytes

Astrocytes perform multiple homeostatic functions in the CNS; they regulate the plasticity of synapses and synthesis of neurotransmitters (Ullian et al., 2004; Volterra and Meldolesi, 2005; Sloan and Barres, 2014), they maintain the blood brain barrier, and they provide neurotrophic support to MNs by releasing glial-derived neurotrophic factor (GDNF) and transforming growth factor β1 (TGF-β1) amongst others. Like microglia, during the neurodegenerative process, astrocytes can exist in two states, either reactive or activated, and activated astrocytes lose their neuroprotective functions and become neurotoxic during disease (Yamanaka et al., 2008; Ilieva et al., 2009; Valori et al., 2014; Das and Svendsen, 2015). Also, like microglia, astrocytes are implicated in the progression rather than onset of ALS. Deletion of SOD1 from astrocytes slowed disease progression, but not disease onset, in SOD1^G93A^ mice (Yamanaka et al., 2008; Wang L. et al., 2011), whereas deletion of mutant SOD1 from MNs did delay onset (Boillée et al., 2006a; Wang L. et al., 2009). Furthermore, gene expression changes in MNs, astrocytes and oligodendrocytes start just before disease onset in SOD1^G37R^ mice, but these alterations are first observed in MNs (Sun et al., 2015). Recently, two different subsets of reactive astrocytes were described in the adult CNS, A1 and A2 (Liddelow et al., 2017; Clarke et al., 2018; Miller, 2018) and the A1 reactive astrocytes were associated with the death of both neurons and oligodendrocytes (Liddelow et al., 2017).

There is increasing evidence that astrocytes mediate MN degeneration via the release of neurotoxic factors. Soluble toxic compounds produced by astrocytes expressing mutant SOD1 trigger the selective loss of spinal MNs (Nagai et al., 2007), but not spinal GABAergic neurons, consistent with the specific vulnerability of these cells in ALS (Nagai et al., 2007). Astrocytes in the ventral spinal cord can be distinguished from astrocytes in the dorsal spinal cord by expression of semaphorin A3 (Sema3a), which is implicated in the specific vulnerability to FF-MNs in ALS (see section “Neuroprotective and Neurotoxic Factor Expression in MN Subpopulations” below). Furthermore, astrocytes are also implicated in MN loss and disease progression by mediating AMPA receptor-induced excitotoxicity via EAAT2/GLT-1, as discussed below (section “Neuronal Excitability”). Expression of mutant TDP-43^M337V^ in rat astrocytes led to down-regulation of neurotrophic genes, up-regulation of neurotoxic genes and progressive MN degeneration (Tong et al., 2013; Huang et al., 2014). Conditioned medium from primary astrocyte cultures of SOD1^G86R^ and TDP-43^A315T^ mice also induces MN death through activation of sodium channels and nitro-oxidative stress (Rojas et al., 2014). Furthermore, astrocytes expressing mutant FUS^R521G^ trigger MN death by secreting pro-inflammatory tumor necrosis factor (TNF)-α (Kia et al., 2018). SOD1^G93A^ aggregates in astrocytes appear in late disease stages, selectively in regions with extensive neuronal degeneration and prominent astrogliosis (Jaarsma et al., 2008). This raises the possibility that astroglial aggregate formation is triggered by MN degeneration, implying that disease may spread from neurons to glia (Jaarsma et al., 2008; Sun et al., 2015).

Together these studies suggest the involvement of astrocytes in the selective degeneration of MNs in ALS. Under normal conditions, astrocytes may be able to cope with the expression of low levels of misfolded proteins, but, during cell stress or in the context of MN degeneration, they become more vulnerable, and release factors toxic to MNs, thus producing a vicious cycle. However, the relative resistance of neuronal populations surrounded by reactive astrocytes indicates that the vulnerability of MNs is also determined by cell-autonomous components, such as their genetic background and transcriptional/translational profiles (Boillée et al., 2006a; Sun et al., 2015).

#### Oligodendrocytes and Schwann Cells

The two glial cell types responsible for myelination of axons have also been investigated in the context of ALS. Oligodendrocytes myelinate axons in the CNS whereas Schwann cells are responsible for myelination in the peripheral nervous system (PNS). Whilst they perform similar functions, there are also important differences between these two cell types. Schwann cells form a single myelin sheath around one single axon, whereas oligodendrocytes myelinate many different axons. Furthermore, there are differences in the protein composition of CNS and PNS myelin.

In ALS, TDP-43 pathology has been detected in oligodendrocytes in the motor cortex and spinal cord of both SALS and FALS patients (Arai et al., 2006; Mackenzie et al., 2007; Tan et al., 2007; Zhang et al., 2008; Seilhean et al., 2009; Murray et al., 2011; Philips et al., 2013). In addition, FUS forms cytoplasmic aggregates in oligodendrocytes from ALS patients bearing FUS^R521C^ or FUS^P525L^ mutations (Mackenzie et al., 2011). Degeneration of oligodendrocytes and their precursors was also linked with axon demyelination in both SALS and FALS patients (Kang et al., 2013). In SOD1^G93A^ mice, oligodendrocyte loss in the spinal cord occurs before symptoms appear and importantly, before MN loss, implying that oligodendrocytes are associated with disease onset. This MN loss increases with disease progression, resulting in MNs with only partially myelinated axons in SOD1^G93A^ mice and SOD1^G93A^ rats (Niebroj-Dobosz et al., 2007; Kang et al., 2013; Philips et al., 2013). Whilst the proliferation of oligodendrocyte precursors may compensate for this loss, newly synthetized oligodendrocytes failed to mature and remain dysfunctional in SOD1^G93A^ mice (Magnus et al., 2008; Philips et al., 2013). Recently, SOD1^G85R^ was able to transfer from MNs to nearby oligodendrocytes (Thomas et al., 2017). The selective removal of mutant SOD1 from NG2+ oligodendrocyte progenitors, but not mature oligodendrocytes in SOD1^G37R^ mice, leads to delayed disease onset and prolonged survival (Kang et al., 2013), further suggesting that mutant SOD1-induced oligodendrocyte defects are detrimental to MNs in ALS.

Schwann cells are required for the long-term maintenance of synapses at the NMJ (Reynolds and Woolf, 1992; Son and Thompson, 1995; Reddy et al., 2003). Early studies demonstrated that myelin is altered along peripheral nerves in ALS patients, implying that Schwann cells are involved in disease (Perrie et al., 1993). However, unlike the other glial cell types, more recent studies on the role of Schwann cells in ALS have reached conflicting conclusions. Knockdown of SOD1^G37R^ within Schwann cells significantly accelerates disease progression, concomitant with a specific reduction in insulin-like growth factor (IGF-I), which is protective to MNs (see section “Neuroprotective and Neurotoxic Factor Expression in MN Subpopulations” below) (Lobsiger et al., 2009). This surprising finding, implying that SOD1^G37R^ is protective in Schwann cells, could be linked to the dismutase activity of SOD1. Whereas SOD1^G37R^ retains its enzymatic activity, SOD1^G85R^ does not, and similar experiments performed in SOD1^G85R^ mice resulted in opposite findings; Schwann cell specific knock-down of SOD1^G85R^ delayed disease onset and extended survival (Wang et al., 2012). Furthermore, TGF-β1 produced by Schwann cells promotes synaptogenesis by increasing nerve-muscle contacts (Feng and Ko, 2008), in contrast to TGF-β1 expression in astrocytes which accelerates disease progression in SOD1 mice (Endo et al., 2015). Hence, the role of Schwann cells in ALS remains unclear.

## Intrinsic Factors Specific to MN Subpopulations

Multiple cellular pathways are now implicated in the etiology of ALS, but it remains unclear how dysfunction of these diverse processes can result in the same disease phenotype. Furthermore, the same genetic mutation can result in either ALS, FTD or both conditions, implying that specific disease modifiers exist. Studies using *in vivo* and *in vitro* models of FALS suggest that the intrinsic properties of MNs are crucial for degeneration and/or protection (Boillée et al., 2006a). Importantly, resistant MN subtypes appear to display diverse gene expression profiles from susceptible MNs. Microarray analysis and laser capture microdissection of MNs isolated from oculomotor/trochlear nuclei, the hypoglossal nucleus and the lateral column of the cervical spinal cord in SOD1^G93A^ rats (Hedlund et al., 2010), or in human brain and spinal cords (Brockington et al., 2013), have revealed marked differences between these subpopulations. Importantly, many of the genes that were differentially expressed encode proteins that function in pathways implicated in ALS pathogenesis, such as ER function, calcium regulation, mitochondrial function, ubiquitination, apoptosis, nitrogen metabolism, transport and cellular growth. Interestingly, oculomotor neurons possess a specific and relatively conserved protein signature between humans and rodents, implying that this contributes to the relative resistance of these MNs in ALS/FTD (Hedlund et al., 2010; Comley et al., 2015). Several of these proteins are known to be protective against MN neurodegeneration, such as insulin-like growth factors (IGF) and their receptors (see section “Neuroprotective and Neurotoxic Factor Expression in MN Subpopulations” below). Similarly, other genes highly expressed in vulnerable MNs are implicated in their susceptibility to degeneration, such as semaphorin A3 (Sema A3) and matrix metalloproteinase 9 (MMP-9) (see section “Neuroprotective and Neurotoxic Factor Expression in MN Subpopulations” below). Recently, a comprehensive bioinformatics meta-analysis of ALS modifier genes was performed from 72 published studies (Yanagi et al., 2019). A total of 946 modifier genes were identified and of these, 43 genes were identified as modifiers in more than one ALS gene/model. These included TDP-43, SOD1, ATXN2 and MMP9. Intrinsic factors in MNs might therefore underlie their relative vulnerability or resistance to neurodegeneration in ALS. The two pioneering studies linking gene expression differences to MN vulnerability in ALS (Hedlund et al., 2010; Brockington et al., 2013) have led to several subsequent reports, where the role of specific genes were examined further (summarized in Table 7, and discussed further in the sections below). However, it is also possible that the differences in gene expression reflect the diverse embryological origins or milieu of resistant and susceptible MN groups, or simply the structural and functional differences between oculomotor units and motor units of other skeletal muscles. To date, no studies have extensively characterized the specific transcriptional profile of vulnerable vs. susceptible MNs in TDP-43, C9orf72 FUS or other models of ALS, similar to those performed in SOD1^G93A^ mice and ALS patients (Hedlund et al., 2010; Brockington et al., 2013).

**Table 7:** Table with genes (described in this review) which are differently expressed among neuron subpopulations.

Gene	Gene acronym	Motor neurons						
			
		Cortical	Oculomotor	Onuf’s	Hypoglossal	Slow spinal cord	Fast spinal cord	References
			
		Vulnerable	Resistant	Resistant	Vulnerable	Resistant	Vulnerable	
Insulin-like growth factor I receptor	*IGF-IR*		+			– (cervical spinal MNs)		Allodi et al., 2016
Insulin-like growth factor II	*IGF-II*		+			–		Hedlund et al., 2010; Allodi et al., 2016
Glial cell line-derived neurotrophic factor receptor subunit	*GFRα1*							Shneider et al., 2009
Semaphorin A3	*SemaA3*	+					+ (FF)	De Winter et al., 2006
Na^+^/K^+^ATPase-alpha3						–	+	Ruegsegger et al., 2016
AMPA receptor GluR2 subunits	*GluR2*		+					Brockington et al., 2013
calbindin-D28K	*CaBP*	–	+	+				Alexianu et al., 1994
Parvalbumin		–	+					Alexianu et al., 1994
Calreticulin	*CRT*						–	Bernard-Marissal et al., 2012
matrix metalloproteinase-9	*MMP-9*		–			–	+	Kaplan et al., 2014
Binding immunoglobulin protein co-chaperone	*SIL-1*					+	– (FF)	Filézac de L’Etang et al., 2015
Dynein			–		+	+ (spinal MNs)		Comley et al., 2015


*(+), upregulation; (–), downregulation; gray, unknown.*

In addition to alterations in gene expression profiles, it is also possible that the resistant MNs in ALS display differing functional or morphological properties to those more susceptible to degeneration. A recent study demonstrated that cultures obtained from surviving MNs of SOD1^G93A^ mice displayed more dendritic branching and axonal outgrowth, as well as increased actin based-growth cones, implying that they have more regenerative capacity (Osking et al., 2019).

### RNA Homeostasis

Abnormal RNA homeostasis is increasingly implicated in the pathophysiology of ALS/FTD, consistent with the functions of TDP-43 and FUS in regulating RNA splicing and transport (Polymenidou et al., 2011; Tank et al., 2018). In the transgenic SOD1^G93A^ rat, differences in the number of genes involved in transcription, RNA metabolism, RNA binding and splicing, and regulation of translation, were evident between neuronal populations located in the oculomotor/trochlear nucleus, the hypoglossal nucleus and the lateral column of the cervical spinal cord (Hedlund et al., 2010). These results therefore suggest that RNA homeostatic processes are involved in the differential vulnerability of specific subtypes of MNs in ALS. However, further studies in this area are required to investigate this possibility, particularly in relation to TDP-43 and FUS.

### Neuroprotective and Neurotoxic Factor Expression in MN Subpopulations

Differential expression of pro-survival or toxic factors is also implicated in the specific vulnerability of MN subtypes. The IGFs are proteins with high homology to insulin that form part of the IGF “axis” that promotes cell proliferation and inhibits apoptosis. In the normal rat, IGF-I is highly expressed in oculomotor neurons, where it is protective against glutamate-induced toxicity (Hedlund et al., 2010; Allodi et al., 2016). This may be due to activation of the PI3K/Akt and p44/42 MAPK pathways, which both inhibit apoptosis (Siddle et al., 2001; Sakowski et al., 2009). In addition, its associated receptor, IGF-I receptor (IGF-IR), is also highly expressed in oculomotor neurons and on the extraocular muscle endplate (Allodi et al., 2016). IGF-IR is important for the survival of neurons following hypoxic/ischemic injury (Vincent and Feldman, 2002; Liu et al., 2011) by upregulation of neuronal cellular inhibitor of apoptosis-1 (cIAP-1) and X-linked inhibitor of apoptosis (XIAP) (Liu et al., 2011). Delivery of IGF-II using AAV9 to the muscle of mutant SOD1^G93A^ mice extended life-span by 10%, prevented the loss of MNs and induced motor axon regeneration (Allodi et al., 2016). These findings indicate that differential expression of IGF-II and IGF-IR in oculomotor neurons might contribute to their relative resistance to degeneration in ALS/FTD.

Conversely, aberrant expression of axon repulsion factors near the NMJ may contribute to neurodegeneration in ALS. Sema3A and its receptor neuropilin 1 (Nrp1) are involved in axon guidance during neural development (Huber et al., 2005; Moret et al., 2007). Sema3A is specifically upregulated in terminal Schwann cells near NMJs of vulnerable FF muscle fibers in mutant SOD1^G93A^ mice (De Winter et al., 2006). Nrp1 is upregulated in axon terminals of the NMJ in this model and administration of an antibody against the Sema3A-binding domain of Nrp1 delayed the decline of motor functions while prolonging the lifespan of SOD1^G93A^ mice (Venkova et al., 2014). Furthermore, Sema3A is upregulated in the motor cortex of ALS patients (Körner et al., 2016; Birger et al., 2018), but not in the spinal cord. Sema3A induces death of sensory, sympathetic, retinal and cortical neurons (Shirvan et al., 2002; Ben-Zvi et al., 2008; Jiang et al., 2010; Wehner et al., 2016), but not spinal neurons (Molofsky et al., 2014; Birger et al., 2018). Similarly, Sema3A induces apoptosis of human cortical neurons but promotes survival of spinal MNs (Birger et al., 2018). Furthermore, loss of Sema3A-expressing astrocytes in the ventral spinal cord leads to selective degeneration of α-MNs, but not γ-MNs (Hochstim et al., 2008; Molofsky et al., 2014). These data indicate that whilst Sema3A and Nrp1 contribute to the loss of MNs in ALS, some neuronal subpopulations are more susceptible than others. There is also evidence that other axon guidance proteins are associated with the susceptibility of MNs in ALS. Increased expression of ephrin A1 has been demonstrated in the vulnerable spinal MNs of ALS patients (Jiang et al., 2005). *EPHA4*, which is a disease modifier in zebrafish, rodent models and human ALS, encodes an Eph receptor tyrosine kinase, which is involved in axonal repulsion during development and in synapse formation, plasticity and memory in adults (Van Hoecke et al., 2012). The more vulnerable MNs express higher levels of EPHA4, and neuromuscular re-innervation is inhibited by Epha4. In ALS patients, EPHA4 expression also inversely correlates with disease onset and survival (Van Hoecke et al., 2012).

Matrix Metalloproteinase (MMP9) has been recently identified as another determinant of selective neuronal vulnerability in SOD1^G93A^ mice (Kaplan et al., 2014). MMP-9 was strongly expressed by vulnerable FR spinal MNs, but not oculomotor, Onuf’s nuclei or S α-MNs, and it enhanced ER stress and mediated muscle denervation in this model (Kaplan et al., 2014). Delivery of MMP-9 into FF-MNs, but not in oculomotor neurons, accelerates denervation in SOD1^G93A^ mice (Kaplan et al., 2014). Similarly, another study demonstrated that reduction of MMP-9 expression attenuated neuromuscular defects in rNLS8 mice expressing cytoplasmic hTDP43^ΔNLS^ in neurons (Spiller et al., 2019). Edaravone, a free radical scavenger which inhibits MMP-9 expression, was recently approved for the treatment of ALS in Japan, South Korea, United States and Canada (Yoshino and Kimura, 2006; Ito et al., 2008; Yagi et al., 2009). Further molecular investigations into the differences and similarities between different motor units in ALS should yield additional insights into their vulnerability to neurodegeneration.

Polymorphisms in specific genes have also been linked to MN vulnerability. In SALS patients, variants in the gene encoding *UNC13A* are associated with greater susceptibility to disease and shorter survival (Diekstra et al., 2012). UNC13A functions in vesicle maturation during exocytosis and it regulates the release of neurotransmitters, including glutamate. Mutations in *EPHA4* are also associated with longer survival (Van Hoecke et al., 2012), implying that Epha4 modulates the vulnerability of MNs in ALS. Furthermore, repeat expansions in the gene encoding ataxin 2 (*ATXN2*), which cause spinocerebellar ataxia type 2 (SCA2), are also increased in ALS patients compared to healthy controls (Ross et al., 2011). This implies that *ATXN2* repeat expansions are also related to MN vulnerability to neurodegeneration in ALS.

### Neuronal Excitability

The excitability properties of MNs are also implicated in the selective degeneration of specific MN subtypes in ALS. Alterations in MN excitability have been reported during the asymptomatic disease stage in the SOD1^G93A^ (Saxena et al., 2013), s-SOD1^G93A^ (Pambo-Pambo et al., 2009) and SOD1^G85R^ (Bories et al., 2007) mouse models, in iPSC-derived MNs (Vucic et al., 2008; Wainger et al., 2014) and in SALS and FALS patients (Vucic and Kiernan, 2010; Devlin et al., 2015). Specific isoforms of the sodium–potassium pump (Na^+^/K^+^ATPase), which generates the Na^+^/K^+^ gradients that drive the action potential, are associated with the specific vulnerability of MN subtypes. Misfolded mutant SOD1 forms a complex with the α3 isoform of Na^+^/K^+^ATPase, and this leads to impairment in its ATPase activity. Altered levels of this isoform were also observed in spinal cords of SALS and non-SOD1 FALS patients (Ruegsegger et al., 2016). Importantly, α3 is the major isoform in vulnerable FF-MNs, whereas both α1 and α3 predominate in FR-MNs, and S-MNs express only α2. Furthermore, viral-mediated expression of a mutant Na^+^/K^+^ATPase-α3 that cannot bind to mutant SOD1 restored Na^+^/K^+^ATPase-α3 activity, delayed disease manifestations and increased lifespan in two different mutant SOD1 mouse models (SOD1^G93A^ and SOD1^G37R^) (Ruegsegger et al., 2016). This indicates that modulating the activity of the α3 isoform of the Na^+^/K^+^ATPase, and therefore modulating the excitability status of MNs, is important in neurodegeneration in ALS.

However, increasing MN excitability is also neuroprotective to MNs in ALS. Enhancing MN excitability by delivering AMPA receptor agonists to mutant SOD1^G93A^ mice reversed misfolded mutant protein accumulation, delayed pathology and extended survival, whereas reducing MN excitability by antagonist CNQX accelerated disease and induced early denervation, even in the more resistant S-MNs (Saxena et al., 2013). However, MN subpopulations can be differentially affected by changes in excitability. Disease resistant S-MNs exhibit hyper-excitability in ALS patients (de Carvalho and Swash, 2017) and early in disease in mutant SOD1^G93A^ mice, whereas disease vulnerable FF-MNs are not hyper-excitable, again highlighting increased excitability as a protective property in ALS (Leroy et al., 2014). Also, the vulnerable masticatory trigeminal MNs from SOD1^G93A^ mice exhibit a heterogeneous discharge pattern, unlike oculomotor neurons (Venugopal et al., 2015). However, MNs in FALS and SALS patients are hyperexcitable early in disease course, but then later become hypo-excitable (Vucic et al., 2008; Menon et al., 2015), indicating that modulation of neuronal excitability is a factor influencing the development of ALS.

### Excitotoxicity

Excitotoxicity is the process by which neurons degenerate from excessive stimulation by neurotransmitters such as glutamate, due to overactivation of NMDA or AMPA receptors. This can result from pathologically high levels of glutamate, or from excitotoxins like NMDA and kainic acid, which allow high levels of Ca^2+^ to enter the cell. One line of evidence supporting a role for excitotoxicity in ALS is that riluzole, one of the only two drugs available for ALS patients, has anti-excitotoxic properties (Bensimon et al., 1994; Lacomblez et al., 1996). Riluzole inhibits the release of glutamate due to inactivation of voltage-dependant Na^+^ channels on glutamatergic nerve terminals (Doble, 1996). Previous studies have suggested that MNs that are less susceptible to excitotoxicity are less prone to degenerate (Hedlund et al., 2010; Brockington et al., 2013).

Ca^2+^ enters neurons through ligand-gated channels or voltage-gated channels such as the voltage-gated-L-type Ca^2+^ channel (Cav1.3), which mediates the generation of persistent inward currents (Xu and Lipscombe, 2001). Cav1.3 is differentially expressed in MN subtypes, with more in the spinal cord compared to the oculomotor and hypoglossal nuclei (Shoenfeld et al., 2014). This Ca^2+^ inward current increases early in disease course in MNs of SOD1^G93A^ mice, which is associated with an increase in Cav1.3 expression.

In addition, the presence of atypical AMPA receptors in MNs compared to other neurons might render them more permeable to Ca^2+^. Functional AMPA receptors normally form a tetrameric structure composed, in various combinations, of the four subunits, GluR1, GluR2, GluR3, and GluR4. The Ca^2+^ conductance of these receptors differs markedly depending on whether GluR2 is a component of the receptor. However, in MNs, AMPA receptors express proportionately fewer GluR2 subunits relative to other types (Kawahara et al., 2003; Sun et al., 2005), which may render them more permeable to Ca^2+^ and thus more vulnerable to excitotoxic injury than other cells. Consistent with this notion, more GluR1 and GluR2 subunits are present in oculomotor neurons compared to spinal MNs in humans (Brockington et al., 2013), and treatment with AMPA/kainate of slice preparations from the rat lumbar spinal cord and midbrain results in more Ca^2+^ influx in spinal cord MNs compared to oculomotor neurons (Brockington et al., 2013). MNs in culture or *in vivo* are selectively vulnerable to glutamate receptor agonists, particularly those that stimulate AMPA receptors and induce excitotoxicity (Carriedo et al., 1996; Urushitani et al., 1998; Fryer et al., 1999; Van and Robberecht, 2000), whereas NMDA does not damage spinal cord MNs (Curtis and Malik, 1985; Pisharodi and Nauta, 1985; Hugon et al., 1989; Urca and Urca, 1990; Nakamura et al., 1994; Ikonomidou et al., 1996; Kruman et al., 1999). Moreover, ALS-vulnerable α-spinal cord MNs display greater AMPA receptor current density than other spinal neurons (Vandenberghe et al., 2000). Furthermore, when this density is reduced pharmacologically to levels similar to spinal neurons, these MNs are no longer vulnerable to activation of AMPA receptors. Similarly, when mutant SOD1^G93A^ mice are crossed with mice overexpressing the GluR2 subunit in cholinergic neurons, the resulting progeny possess AMPA receptors with reduced permeability to Ca^2+^ and prolonged survival compared to SOD1^G93A^ mice (Tateno et al., 2004), highlighting the importance of AMPA receptors and GluR2 in ALS.

Editing of mRNA controls the ability of the GluA2 subunit to regulate Ca^2+^-permeability of AMPA receptors. RNA editing is a post-transcriptional modification (Gln; Q to Arg; R) in the GluA2 mRNA, and the AMPA receptor is Ca^2+^-impermeable if it contains the edited GluA2(R) subunit. Conversely, the receptor is Ca^2+^-permeable if it lacks GluA2 or if it contains the unedited GluA2(Q) subunit. Interestingly, spinal MNs in human ALS patients display less GluR2 Q/R site editing (Kawahara et al., 2004; Aizawa et al., 2010). GluR2 pre-mRNA is edited by the enzyme adenosine deaminase isoform 2 (ADAR2) (Kortenbruck et al., 2001) and reduced ADAR2 activity correlates with TDP-43 pathology in human MNs (Aizawa et al., 2010). Furthermore, when ADAR2 is conditionally knocked-down in MNs in mice, a decline in motor function and selective loss of MNs in the spinal cord and cranial motor nerve nuclei was observed (Hideyama et al., 2012). In contrast, MNs in the oculomotor nucleus were retained, despite a significant decrease in GluR2 Q/R site editing (Hideyama et al., 2010). Notably, cytoplasmic mislocalization of TDP-43 was present in the ADAR2-depleted MNs (Yamashita et al., 2012) and TDP-43 was also localized at the synapse, further highlighting a link between ADAR2, GluR2 and TDP-43 (Wang et al., 2008; Feiguin et al., 2009; Polymenidou et al., 2011; Gulino et al., 2015).

Motor neurons may be vulnerable to excitotoxicity because they possess a lower capacity than other neurons to buffer Ca^2+^ upon stimulation (Van Den Bosch et al., 2006). Several electrophysiological studies have demonstrated that susceptible MNs in ALS have a limited capacity to buffer Ca^2+^ compared to resistant MNs (Lips and Keller, 1998, 1999; Palecek et al., 1999; Vanselow and Keller, 2000). Ca^2+^-binding proteins, such as calbindin D28K and parvalbumin, protect neurons from Ca^2+^-mediated cell death by enhancing Ca^2+^ removal after stimulation (Chard et al., 1993). In human autopsy specimens, both proteins are absent in MN populations lost early in ALS (cortical, spinal and lower cranial MNs), whereas MNs targeted later in disease course (Onuf’s nucleus, oculomotor, trochlear, and abducens MNs) expressed markedly more of each (Alexianu et al., 1994). Similarly, in pre-symptomatic SOD1^G93A^ mice, lower levels of the Ca^2+^ binding ER chaperone calreticulin (CRT) were detected in vulnerable FF-MNs of the tibialis anterior muscle, compared to resistant MNs of the soleus (Bernard-Marissal et al., 2012). Knock-down of CRT *in vitro* was sufficient to trigger MN death by the Fas/NO pathway (Bernard-Marissal et al., 2012). Furthermore, reduced CRT levels and activation of Fas both trigger ER stress and cell death specifically in vulnerable SOD1^G93A^-expressing MNs (Bernard-Marissal et al., 2012). These studies suggest that expression of Ca^2+^-binding proteins may confer resistance to excitotoxic stimuli (Alexianu et al., 1994; Obál et al., 2006). However, overexpression of parvalbumin in high-copy SOD1^G93A^ mice was beneficial (Laslo et al., 2000), although these findings have been challenged (Beers et al., 2001). Also, the loss or reduction of parvalbumin and calbindin D-28k immunoreactivity in large MNs at early stages in SOD1-transgenic mice suggest that these Ca^2+^-binding proteins contribute to the selective vulnerability of MNs (Sasaki et al., 2006). Conversely, parvalbumin levels are significantly less in oculomotor neurons from SOD1^G93A^ mice compared to spinal cord MNs (Comley et al., 2015). Hence, these conflicting data argue against the involvement of Ca^2+^-binding proteins in oculomotor neuron resistance to degeneration. However, together these studies suggest that neuronal excitability and excitotoxicity are determinants of the selective vulnerability of spinal cord neurons, and the relative resistance of oculomotor neurons, in ALS.

### Endoplasmic Reticulum Stress

The ER is responsible for the folding and quality control of virtually all proteins that transit through the secretory pathway. Hence it is a fundamental aspect of proteostasis. Unfolded or misfolded proteins are retained in the ER, which activates the unfolded protein response (UPR). This aims to improve the cellular protein folding capacity by inhibiting translation, upregulating ER chaperones – such as immunoglobulin binding protein (BiP) and protein disulfide isomerase (PDI) – and stimulating protein degradation (Walter and Ron, 2011; Rozas et al., 2017; Shahheydari et al., 2017). Numerous ALS-related proteins chronically active the UPR, including ALS-associated mutant forms of SOD1 (Nishitoh et al., 2008), TDP-43 (Walker et al., 2013), C9orf72 (Dafinca et al., 2016), Vesicle-associated membrane protein-associated protein B (VAPB) (Suzuki et al., 2009) and FUS (Farg et al., 2012). ER stress has also been detected in sporadic ALS patients (Ilieva et al., 2007; Atkin et al., 2008). Furthermore, ER stress is linked to excitability in ALS. Mutant SOD1 induces a transcriptional signature characteristic of ER stress, which also disrupts MN excitability (Kiskinis et al., 2014). Similarly, modulating the excitability properties of human iPSC-derived MNs alters the UPR (Kiskinis et al., 2014). Conversely, treatment of MNs with salubrinal, an inhibitor of ER stress which inhibits eIF2α dephosphorylation (Boyce et al., 2005), reduced the excitability of MNs (Kiskinis et al., 2014). Similar results were obtained in MNs from patients carrying C9orf72 repeat expansions or VCP mutations (Kiskinis et al., 2014; Dafinca et al., 2016; Hall et al., 2017). Moreover, pharmacological reduction of neuronal excitability in SOD1^G93A^ mice specifically reduced BiP accumulation in ipsilateral FALS α-MNs (Saxena et al., 2013). Hence, together these findings indicate that induction of the UPR and the electrical activity of MNs are both closely related in ALS.

An *in vivo* longitudinal analysis of MNs revealed that ER stress influences disease manifestations in SOD1^G93A^ and SOD1^G85R^ mouse models of FALS (Saxena et al., 2009). However, activation of the UPR is detrimental to mutant s-SOD1^G93A^ mice, leading to failure to reinnervate NMJs. Conversely, treatment with salubrinal attenuated axon pathology and extended survival in mutant SOD1^G93A^ mice (Saxena et al., 2009). Initiation of the UPR was detected specifically in FF-MNs in asymptomatic SOD1^G93A^ mice, but not in S-MNs (Saxena et al., 2009). Hence these findings indicate that the more vulnerable MNs develop ER stress first, thus linking the UPR to MN susceptibility in ALS. FF-MNS may be more vulnerable to ER stress because they have much lower levels of BiP co-chaperone SIL1 compared to S-MNs (Filézac de L’Etang et al., 2015). SIL1 is protective against ER stress and reduces the formation of mutant SOD1 inclusions *in vitro*. Conversely SIL1 depletion leads to disturbed ER and nuclear envelope morphology, defective mitochondrial function, and ER stress, thus linking SIL1 to neurodegeneration (Roos et al., 2016). Furthermore, AAV-mediated overexpression of SIL1 in MNs of SOD1^G93A^ mice preserves FF MN axons and prolongs survival by 25–30% compared to littermates (Filézac de L’Etang et al., 2015). In addition, SIL1 levels are reduced in MNs of mutant TDP-43^A315T^ mice, and are increased in the surviving MNs of SALS patients, also implying that SIL1 is protective in ALS (Filézac de L’Etang et al., 2015).

Consistent with these studies, ER stress is present specifically in anterior horn MNs in *knock-in* mice expressing BiP artificially retained in the ER. Furthermore, this was accompanied by the accumulation of ubiquitinated proteins and wild type SOD1 (Mimura et al., 2008; Jin et al., 2014), reminiscent of SALS (Bosco et al., 2010). Significant changes in mRNAs of ER stress genes were also detected in the cerebellum by transcriptome analysis (Prudencio et al., 2015). These studies together link SIL1 and BiP to neurodegeneration in both neuronal subpopulations in ALS/FTD.

PDI is also upregulated in SOD1 mice and human SALS spinal cord tissues (Ilieva et al., 2007; Atkin et al., 2008; Sasaki, 2010; Walker et al., 2010; Chen et al., 2015; Sun et al., 2015). Wild type PDI overexpression and related family member Erp57 are protective *in vitro* in neuronal cells expressing mutant SOD1 (Walker et al., 2010; Jeon et al., 2014; Parakh et al., 2018a). Interestingly, mutations in PDI and Erp57 have been identified in ALS patients, and expression in zebrafish induces motor defects (Woehlbier et al., 2016). Furthermore, the levels of PDI in MNs are lower than in astrocytes and oligodendrocytes in SOD1^G37R^ mice (Sun et al., 2015). This implies that MNs are intrinsically more vulnerable to unfolded protein accumulation than other cell types, which may also contribute to their susceptibility in ALS.

It should also be noted, however, that the ER in neurons (and therefore MNs) is not as well characterized as other cell types. In fact, most studies examining UPR mechanisms have involved non-neuronal cells. Neurons possess extensive ER which is distributed continuously throughout the axonal, dendritic and somatic compartments, implying that neurons make unique demands on the ER compared to other cell types (Ramírez and Couve, 2011). Hence, our current soma-centric view of the ER does not consider its role in neuronal processes and how this might relate to their specific functions. This is particularly true for large neurons, such as MNs with their extended axons. The findings that the most susceptible MNs develop ER stress first implies that the ER in MNs may confer unique susceptibility on these cells compared to other MNs and non-neuronal cells. However, this idea requires validation experimentally.

### Mitochondria and Energy Metabolism

Neurons utilize most of their energy at the synapse, which consumes more than a third of the overall cellular ATP (Harris et al., 2012; Niven, 2016). The properties and types of ion channels expressed in a MN influence the energy required to generate an action potential, and the Na^+^/K^+^ pump is estimated to account for 20–40% of the brain’s energy consumption (Purves et al., 2001). The size and shape of a MN also affects its electrical properties, and the distance over which signals must spread. MNs have particularly high energetic demands, even compared to other neurons. They also have large numbers of NMJs as well as high intracellular Ca^2+^ flux as discussed above.

More than 90% of ATP generation in the CNS occurs via mitochondrial oxidative phosphorylation (Hyder et al., 2013; Vandoorne et al., 2018). Reductions in energy metabolism have been reported in ALS (Vandoorne et al., 2018) and mitochondrial abnormalities, such as swelling and morphological changes, are among the earliest signs of pathology in SOD1^G93A^ and SOD1^G37R^ mice (Wong et al., 1995; Kong and Xu, 1998), FUS^R521C^ rats (Huang et al., 2012; So et al., 2018) and wild type TDP-43 mice (Shan et al., 2010; Xu et al., 2010). Moreover, mitochondrial abnormalities are also present in MNs of ALS patient tissues (Fujita et al., 1996; Sasaki and Iwata, 1996; Swerdlow et al., 1998; Dhaliwal and Grewal, 2000; Sasaki et al., 2007). Furthermore, mutant SOD1 specifically associates with mitochondria and interferes with their function (Liu et al., 2004; Pasinelli et al., 2004; Ferri et al., 2006; Sotelo-Silveira et al., 2009; Vande Velde et al., 2011). Decreased activity of mitochondrial respiratory chain complexes was also present in spinal cord sections (Borthwick et al., 1999) and homogenates (Wiedemann et al., 2002) from ALS patients. Consistent with these findings, genes involved in mitochondrial function were upregulated in rat oculomotor neurons compared to hypoglossal and cervical spinal cord MNs. However, it should be noted that the higher firing rate of the former might confer some resistance to energy imbalance (Hedlund et al., 2010; Brockington et al., 2013).

In vulnerable MNs lacking Ca^2+^-binding proteins calbindin and parvalbumin, Ca^2+^ is largely taken up by mitochondria (Lautenschläger et al., 2013). As a result, extensive mitochondrial transport to the dendritic space is required to maintain Ca^2+^ homeostasis. The normal distribution of mitochondria is also perturbed in ALS patient MNs. Whereas they are depleted in distal dendrites and axons, mitochondria also accumulate in the soma and proximal axon hillock (Sasaki et al., 2007). Disturbed mitochondrial dynamics were also described in MNs in mutant SOD1^G93A^ (De Vos et al., 2007; Sotelo-Silveira et al., 2009; Bilsland et al., 2010; Magrané et al., 2014) and TDP-43^A315T^ (Magrané et al., 2014) mice. In addition, iPSC-derived A4V MNs exhibit disturbances in mitochondrial morphology and motility within the axon (Kiskinis et al., 2014). Similarly, expression of mutant TDP-43 in spinal cord primary neurons leads to abnormal distribution of mitochondria (Wang et al., 2013). Dysfunctional Ca^2+^ uptake by mitochondria may therefore result in elevated intracellular Ca^2+^ levels, thus contributing to neurodegeneration.

Compared to FF-MNs, S-MNs have smaller soma and axons, less dendritic branching, and fewer neuromuscular terminals (Kanning et al., 2010). This results in higher input resistance and therefore less energy is required to initiate an action potential in comparison. Moreover, S-MNs contain more mitochondria compared to FF-MNs (Kanning et al., 2010). These two properties may therefore render FF-MNs more vulnerable to depletion of energy than S-MNs. Indeed, a computational analysis study estimated that the energy requirements of FF-MNs are considerably larger than S-MNs for a similar discharge (Le Masson et al., 2014), rendering the former more sensitive to ATP imbalance. Furthermore, the muscle fiber types associated with FF- and S-MNs differ in their major energy source. The slow twitch muscles use mainly oxidative metabolism, whereas the fast-twitch fibers use glycolysis. Hence, the heightened vulnerability of MN subpopulations may relate to their bioenergetic and morphological characteristics. Both the direct interaction of misfolded ALS mutant proteins with mitochondria and the secondary overload of ion uptake could account for mitochondrial metabolism failure, leading to reduced ATP availability (Israelson et al., 2010).

### Motor Neuron Size

Motor neurons can vary widely in their size and this can impact on their physiological functions. There is also increasing evidence that vulnerability to degeneration is related to MN size. The disease-vulnerable FF-MNs somas are larger than the S-MN resistant types, and they possess larger motor units. Moreover, the size of a MN also correlates inversely with its excitability, discharge behavior, firing rate, recruitment during movement, and vulnerability to degeneration in ALS (Henneman, 1957; Le Masson et al., 2014). The soma of MNs from male SOD1^G93A^ mice is larger than those of wild type male mice (Shoenfeld et al., 2014). Furthermore, a recent study demonstrated that not only are the larger MN subtypes more vulnerable to neurodegeneration in SOD1^G93A^ mice, but MNs also increase in size during disease in multiple regions of the spinal cord. Interestingly, *in silico* modeling predicted that the excitability properties of these cells were also altered (Dukkipati et al., 2018). Hence, MN size may alter during disease progression, and this plasticity may impact on the vulnerability of MN subtypes.

### Oxidative Stress

Oxidative stress arises when reactive oxygen species (ROS) or nitrogen species (RNS) accumulate within cells. This can lead to oxidative modifications and altered functional states of proteins, nucleic acids and lipids. Oxidative stress is linked to neurodegeneration in ALS (Carrí et al., 2003) and oxidation products, such as malondialdehyde, hydroxynonenal, and oxidized proteins, DNA or membrane phospholipids, are elevated in SALS and FALS patients (Shaw et al., 1995; Beal et al., 1997; Ferrante et al., 1997; Bogdanov et al., 2000; Shibata et al., 2001) and mouse models of ALS (Gurney et al., 1994; Andrus et al., 1998; Bogdanov et al., 1998; Hall et al., 1998; Liu et al., 1998, 1999; Rizzardini et al., 2003). Mitochondria damage in ALS has also been attributed to intracellular oxidative stress (Fujita et al., 1996). The normal physiological function of SOD1 is the detoxification of superoxide radicals, although loss of SOD1 function is no longer favored as a disease mechanism in ALS (Saccon et al., 2013). However, mutations in SOD1 increase neuronal vulnerability to oxidative stress (Franco et al., 2013; Tsang et al., 2014). Moreover, in response to elevated ROS, SOD1 relocates from the cytoplasm to the nucleus, where it regulates the expression of oxidative resistance and repair genes (Tsang et al., 2014).

Some neurons exhibit differential vulnerability to oxidative damage. Cerebellar granule and hippocampal CA1 neurons are more sensitive to oxidative stress than cerebral cortical and hippocampal CA3 neurons (Wang X. et al., 2009; Wang and Michaelis, 2010). Hence, it is possible that similar differences in vulnerability to oxidative stress might exist between MN populations. However, this possibility needs to be confirmed experimentally.

### Protein Transport

Efficient intracellular trafficking is required to maintain the structure and function of MNs, particularly because MNs have very long axons that connect the soma with distant synaptic sites [reviewed in De Vos and Hafezparast (2017)]. Disorganization of the neuronal cytoskeleton and inhibition of axonal, ER-Golgi, endosomal and nucleocytoplasmic transport, are now widely reported features of ALS [reviewed in Parakh et al. (2018b) and Burk and Pasterkamp (2019)]. Importantly, defects in trafficking could reduce the supply of components necessary for synaptic and/or somal function, and prevent clearance of waste products from the synapse, together contributing to neurodegeneration in ALS.

The existence of mutations in genes encoding cytoskeletal proteins or the cellular transport machinery highlights the involvement of these processes in ALS/FTD. These include tubulin α4A (Smith et al., 2014a; Perrone et al., 2017), a major component of microtubules, neurofilament heavy chain (Figlewicz et al., 1994), a type of intermediate filament, and profilin-1 (Wu et al., 2012; Dillen et al., 2013; Smith et al., 2014b), which is involved in actin polymerization. Similarly, dynactin-1, involved in axonal transport (Puls et al., 2003; Münch et al., 2004; Münch et al., 2005; Liu et al., 2017) and SCFD1 (Sec1 family domain containing 1), involved in ER to Golgi transport (van Rheenen et al., 2016), are also mutated in a small proportion of patients, further implying that protein transport is impaired in ALS/FTD.

Axonal transport defects may be an important factor underlying the selective vulnerability of MNs or MN subtypes in ALS/FTD. Abnormal accumulation of phosphorylated neurofilaments, mitochondria and lysosomes in the proximal axon of large MNs and axonal spheroids, are present in SALS and FALS patients (Hirano et al., 1984; Corbo and Hays, 1992; Okada et al., 1995; Rouleau et al., 1996; Sasaki and Iwata, 1996). Mutant SOD1 slows both anterograde (Williamson and Cleveland, 1999) and retrograde (Chen et al., 2007; Perlson et al., 2009) axonal transport. Cytoskeletal and motor proteins are differentially expressed in spinal MNs compared to oculomotor neurons. This includes peripherin (Hedlund et al., 2010; Comley et al., 2015), which is also found in ubiquitinated inclusions in the spinal cord of FALS (Robertson et al., 2003) and SALS patients (He and Hays, 2004). Overexpression of peripherin leads to defective axonal transport (Millecamps et al., 2006) and late-onset MN degeneration (Beaulieu et al., 1999), implying that differential expression of peripherin contributes to neurodegeneration.

Axonal transport requires the efficient regulation of both dynein and kinesin molecular motors (Melkov et al., 2016), which mediate transport in the retrograde and anterograde directions respectively. Dynein is differentially expressed in vulnerable and susceptible MNs because higher levels are present in spinal and hypoglossal MNs compared to oculomotor neurons (Ilieva et al., 2008). However, dynein levels were significantly decreased in motor nuclei in SOD1^G93A^ mice compared to wild type mice although its expression in MNs was equivalent (Comley et al., 2015). Similar patterns were observed in ALS patients (Comley et al., 2015). Disruption of dynein inhibits axonal transport and results in abnormal redistribution of mitochondria (Varadi et al., 2004) and late-onset degeneration in mice (LaMonte et al., 2002). Several FALS-linked SOD1 mutants co-localize with dynein/dynactin *in vitro* and SOD1^G93A^ mice (Ligon et al., 2005; Zhang et al., 2007; Shi et al., 2010), which perturbs axonal transport and synaptic mitochondrial content (De Vos et al., 2007). The lower expression of dynein in oculomotor neurons might therefore confer resistance to axonal transport defects in ALS. However, it is also possible that this simply reflects less need for retrograde transport in oculomotor neurons due to their smaller cell bodies, shorter axons and lower requirements for energy, compared to spinal and hypoglossal MNs. Nevertheless, the inefficient axonal transport of mitochondria may confer loss of energy at the synapse in vulnerable MN subpopulations. These MNs require more energy to function than other cells, leading to disturbed synaptic activity.

Kinesin-dependant axonal transport is also disrupted in ALS. Oxidized forms of wild type SOD1 immunopurified from SALS tissues inhibited kinesin-based fast axonal transport (Bosco et al., 2010). However, no interaction between members of the kinesin family (KIF5A, 5B or 5C) and SOD1 was detected in SOD1^G93A^ mice. High expression of KIF proteins is also associated with neurodegeneration. KIF5C was abundantly expressed in vulnerable spinal MNs in SOD1^G93A^ mice (Kanai et al., 2000), but a marked reduction in KIF3Aβ levels was detected in the motor cortex of SALS patients (Pantelidou et al., 2007). Furthermore, reduced kinesin-associated protein 3 (KIFAP3) expression was linked to an increase in the survival of ALS patients (Landers et al., 2009) and changes in the transport of choline acetyltransferase transporter (ChAT) along axons. KIF5C is expressed more in rat spinal MNs than oculomotor and hypoglossal MNs (Hedlund et al., 2010), However, further work is necessary to determine if this is related to ALS, and to examine whether KIFs are differentially expressed in neuronal subtypes.

Defects in the secretory pathway are also linked to ALS. Depletion of TDP-43 inhibits endosomal trafficking and results in lack of neurotrophic signaling and neurodegeneration (Schwenk et al., 2016). Similarly, inhibition of the first part of the classical secretory pathway, ER-Golgi transport, is also induced by mutant SOD1, TDP-43 and FUS (Sundaramoorthy et al., 2013; Soo et al., 2015). This mechanism has been described as a possible trigger for ER stress (Soo et al., 2015), which, as detailed above, is linked to neuronal susceptibility. Both endosomal and ER-Golgi transport are also linked to transport within the axon. However, it remains to be determined if these other forms of trafficking are directly associated with selective neuronal susceptibility in ALS.

Defective nucleocytoplasmic transport is emerging as an important cellular mechanism in the initiation or progression of ALS. Nuclear pore pathology is present in the brain of SALS and C9orf72 patients (Zhang K. et al., 2015; Chou et al., 2018). C9orf72 repeat expansions impair protein trafficking from the cytoplasm to the nucleus, and reduce the proportion of nuclear TDP-43 in patient-derived MNs (Zhang K. et al., 2015), thereby mimicking the nuclear depletion of TDP-43 in ALS patients (Neumann et al., 2006). Proteins involved in nucleocytoplasmic transport are abnormally localized in aggregates in the cortex of C9orf72 ALS patients, patient-derived MNs and the brain of C9orf72 mouse models (Zhang K. et al., 2015, Zhang et al., 2016). Similarly, TDP-43 pathology disrupts nuclear pore complexes and lamina morphology in cell lines and patient-derived MNs. Furthermore, insoluble TDP-43 aggregates also contain components of the nucleocytoplasmic machinery (Chou et al., 2018). Both protein import and RNA export were impaired by mutant TDP-43 in the brain of SALS mouse primary neurons (Chou et al., 2018). A recent meta-analysis of ALS modifier genes identified several genes encoding proteins involved in nucleocytoplasmic shuttling (Yanagi et al., 2019). In fact, the most enriched gene ontology term in this study was “protein import into the nucleus,” and it included KPNB1, encoding importin subunit beta-1, which was identified as a genetic modifier in three separate ALS models. Interestingly, the gene encoding lamin B1 subunit 1, which is involved in nuclear stability, was upregulated in oculomotor neurons compared to hypoglossal MNs and spinal cord MNs (Hedlund et al., 2010). Furthermore, lamin B1 is also known to possess cellular protective functions such as controlling the cellular response to oxidative stress (Malhas et al., 2009), DNA repair (Butin-Israeli et al., 2015) and RNA synthesis (Tang et al., 2008). It is therefore tempting to speculate that lamin B1 confers resistance to specific MN populations when highly expressed. However, further work is necessary to examine this possibility.

## Aging

Although genetic mutations are present throughout life, ALS most commonly develops in mid-adulthood (50–60 years), implying that the normal aging process renders MNs vulnerable to degeneration. However, there is considerable variability in disease progression amongst mutation carriers, even within the same families. Hence, this implies that there is no simple correlation between genetics and disease phenotypes, suggesting that environmental factors and the normal aging process are relevant to understand neuronal vulnerability in ALS/FTD.

Aging results in the accumulation of detrimental biological changes over time. The reduction of muscle mass and strength (sarcopenia) is one of the major causes of disability in older persons (Enoka et al., 2003; Lauretani et al., 2003; Delmonico et al., 2009; Clark and Manini, 2012), which affects gait speed, balance, and the command of fine motor skills (Fried et al., 2004; Sorond et al., 2015). The deterioration of motor functions with advancing age therefore increases the risk of injury and age-associated diseases such as ALS/FTD (Spiller et al., 2016b; Niccoli et al., 2017).

Aging-associated muscle weakness also results from impairment of the activity of MNs contacting skeletal muscles (Fiatarone and Evans, 1993; Manini et al., 2013). High resolution structural MRI imaging reveals prominent atrophy in the primary motor cortex (Salat et al., 2004), as early as middle life in humans. Age-related decreases in white matter mass and myelinated nerve fiber length also correlate with reductions in the size of the motor cortex (Marner et al., 2003). However, loss of neurons during normal human aging is restricted to specific regions of the CNS only, and the number of cells lost is only slight, contrary to previous convictions that significant loss of neurons occur in the human cortex (Pannese, 2011). Instead, age-related changes observed in aged rhesus monkeys and mice appear to involve loss of dendrites and axons, and demyelination, resulting in significant loss of synapses without loss of the neuronal soma (Pannese, 2011). Similarly, there are fewer cholinergic and glutamatergic synaptic inputs directly abutting α-MNs in aged animals, indicating that aging causes α-MNs to shed synaptic inputs. Thus, both impairment of axon function and substantial loss of synaptic inputs may contribute to age-related dysfunction of α-MNs, without loss of the soma (Maxwell et al., 2018). As a consequence, motor units are gradually lost over the first six decades of life, and this accelerates thereafter (Deschenes, 2011). These studies together indicate that neuronal atrophy and axonal impairment, with reduced neuromuscular activity in the absence of MN loss, occur with normal aging.

A major component of aging-related muscle weakness is breakdown in communication between the brain and NMJ. This is related to increased neural noise which reduces the accuracy of neural transmission (Manini et al., 2013). This can result in activation of the motor unit, so that it becomes erratic, and together with diminished glutamate uptake into MNs, leads to an inability to exert muscle force and motor control (Manini et al., 2013). Furthermore, susceptibility of neurons to cellular stress, due to impairment of proteostasis and/or increased oxidative or metabolic stress during normal aging, may render MNs vulnerable to degeneration. Hence, genetic and environmental factors may combine to determine whether a MN can withstand an age-related disease such as ALS or not (Mattson and Magnus, 2006).

### Age-Related Proteostasis Disturbance

During the aging process, a decline in the normal cellular ability to maintain proteostasis is observed and, as a result, damaged proteins accumulate (Kikis et al., 2010). Thus the normal aging process in MNs that are already weakened by ALS-associated insults, such as the presence of misfolded proteins or environmental factors, may combine to induce neurodegeneration. MN populations that are more susceptible in ALS may therefore be less able to tolerate disturbances in proteostasis than the more resistant populations (Neumann et al., 2006; Kikis et al., 2010).

Mitochondria play a crucial role in neuronal aging. Normal features observed in the aging brain include the accumulation of mutations in mitochondrial DNA, the production of ROS, mitochondrial metabolic abnormalities and altered Ca^2+^ storage (Sun et al., 2016). Remarkably, mitochondria in different regions of the CNS are not equally affected during aging. The sensitivity of the mitochondrial permeability transition pore to Ca^2+^ in the cortex and hippocampus is greater than that of the striatum and the cerebellum in aged rats (LaFrance et al., 2005; Brown et al., 2006). The cellular location of mitochondria is also relevant to the aging processes. Synaptic mitochondria are more prone to oxidative stress-induced damage than mitochondria located in the soma (Brown et al., 2006; Reddy and Beal, 2008). In addition, synaptic mitochondria display a limited capacity to accumulate Ca^2+^, unlike those located in the soma (Brown et al., 2006). Furthermore, marked differences have been described between mitochondria located in the spinal cord and those found in distal axons of MNs from aged rats. In the axon termini at the NMJ, mitochondria swelling, fusion and an abundance of megamitochondria (giant mitochondria) during aging have been reported (García et al., 2013). These studies therefore imply that mitochondria become dysfunctional in aged MNs, which might sensitize vulnerable MN populations to ALS/FTD. Mitochondria located at the synapse may also be particularly vulnerable to these age-related processes.

### Age-Related DNA Damage

The mammalian genome is under constant attack from both endogenous and exogenous sources. This can result in DNA damage, mutations and impaired cellular viability if not repaired correctly (Madabhushi et al., 2014). There is a significant increase in DNA damage during aging due to reduced capacity of DNA repair. Moreover, erroneous repair of DNA lesions can result in further mutations in the aged brain (Vijg and Suh, 2013). DNA damage is increasingly implicated in neurodegenerative disorders, including ALS, where it is induced by the C9orf72 repeat expansion (Farg et al., 2017; Walker et al., 2017). Interestingly, there is also evidence that both FUS and TDP-43 function in the DNA damage response, in either prevention of damage or repair of R loop-associated DNA damage (Hill et al., 2016). In addition, impairment of the DNA damage response due to the presence of ALS/FTD-associated FUS mutations induces neurodegeneration (Higelin et al., 2016; Naumann et al., 2018). It is therefore possible that the normal aging process results in an impaired ability to repair DNA in MNs. This may be an important source of cellular stress that precipitates neurodegeneration in cells already exposed to pathological events throughout life. However, recent work suggests that mutant SOD1^G93A^ does not impact on DNA strand integrity, implying that DNA damage is not present in all forms of ALS (Penndorf et al., 2017).

## Conclusion

**FIGURE 4:**
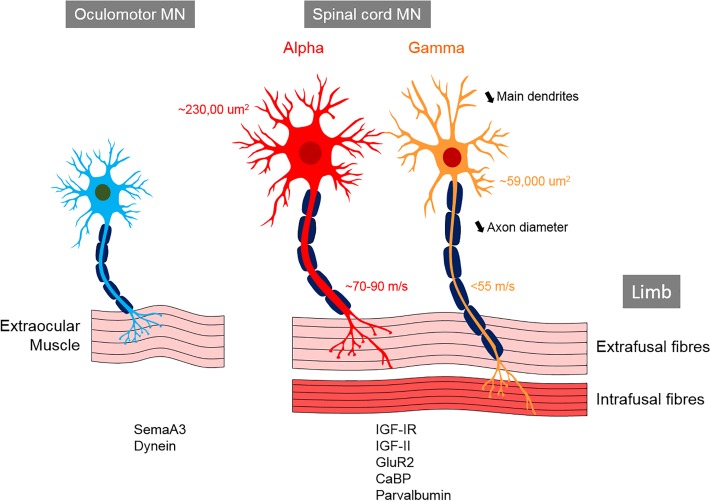
Reported differences between the vulnerable (ventral spinal cord MNs) and resistant (oculomotor) motor neurons in ALS. The surface area and axonal conduction velocities referred to here were obtained from studies in cats (Westbury, 1982). The α-MNs innervate highly contracting extrafusal fibers, whereas γ-MNs innervate intrafusal fibers that contract much less; oculomotor neurons innervate the extraocular muscles in the orbit. α-MNs are larger than γ-MNs and oculomotor neurons and possess more dendritic trees. α-MNs are further subdivided based on their size and function. The proteins listed at the bottom of the figure are those enriched in each MN population.

Motor neurons are unique cells compared to other neurons. They are large cells, with extraordinarily long axons, and very high energetic requirements, which may render them uniquely susceptible to degeneration in ALS. Remarkably, however, not all MNs are equally affected, and there are marked differences in vulnerabilities between MN subtypes, even within the same motor unit. The resistant MNs possess distinct morphological and functional characteristics, as well as different gene expression profiles, compared to the more vulnerable groups (Figure 4). Importantly, the oculomotor neurons continue to function, even in the late stages of ALS when the vulnerable spinal and other MNs are significantly depleted. These oculomotor neurons are anatomically and functionally very different from all other motor units: they are much smaller, and their function involves sensing rather than movement, hence different circuits are involved. In contrast, spinal MNs are more prone to hyperexcitation and they express high levels of AMPA receptors, they are more prone to develop ER stress, and they do not buffer Ca^2+^ as well as the more resistant MN types. These properties may confer unique sensitivity to neurodegeneration in ALS. Interestingly, even within spinal MNs, there are distinct differences in vulnerability, because FF-MNs degenerate first, followed by FR-MNs, and the more resistant S-MNs degenerate later. Similarly, these cells also display differences in excitability and ER stress.

**FIGURE 5:**
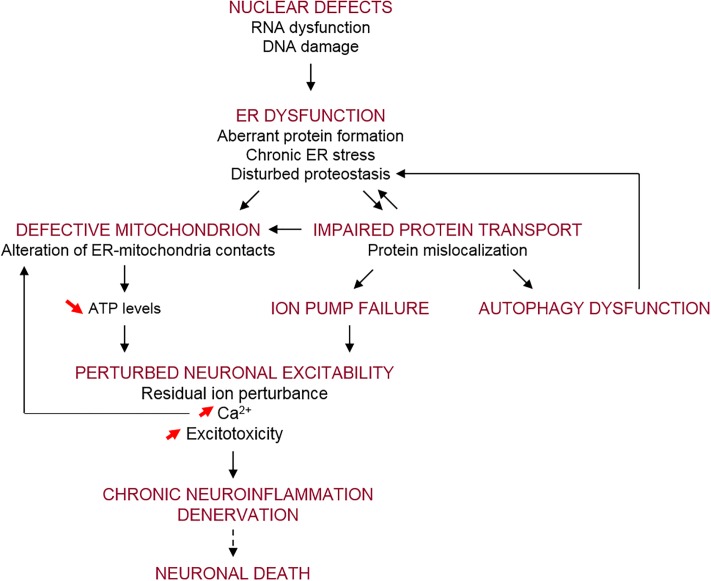
Diagram showing a hypothetic cascade of cellular events leading to neurodegeneration and neuronal death in motor neurons in ALS/FTD. This schematic diagram summarizes the key features occurring in vulnerable MNs. Resistant MNs are protected by the expression of a genes controlling cellular mechanisms that are defective in ALS/FTD (RNA dysfunction, ER stress, mitochondrial defects, protein transport dysfunction, dysregulation of neuronal excitability and excitotoxicity). These processes can be exacerbated by age, environmental and genetic mutations.

A hypothetical model is presented in Figure 5, summarizing the possible molecular mechanisms involved in MN vulnerability in ALS. The regulation of synaptic plasticity and neuronal excitability may underlie susceptibility in ALS involving nuclear-cytoplasmic defects, ER stress, transport dysfunction and mitochondrial alterations. From an initial site of onset, neurodegeneration begins in susceptible MN groups, and then spreads contiguously throughout the neuroanatomy, in a defined pattern, to the surrounding cells. This therefore highlights the role of impaired neurotransmission in triggering and propagating neurodegeneration in ALS. Glial cells are involved in both the onset and progression of ALS.

The susceptibility of specific MN groups, however, is further complicated by the heterogeneous nature of ALS, even within the same families, and the different patterns of motor involvement. Stratification of ALS patients into distinct subtypes and investigations into MNs susceptibilities may reveal more insights why specific groups of MNs degenerate first in ALS in the future. However, the blurring of some neurodegenerative disorders, including ALS and FTD, and the presence of C9orf72 mutations in several other neurodegenerative conditions as well as ALS, is another confounding factor. Understanding the fundamental mechanisms dictating MN vulnerability in ALS is central to our understanding of this devastating disorder. Hence, studies in this area may lead to novel therapeutic insights in the future.

## Author Contributions

MV wrote the “Site-Specific Onset and Spread of Neurodegeneration in ALS” section. MJ wrote the “Role of Glial Cells in Driving Disease Progression” section. SS wrote the “Aging” section. AR conceived and prepared the figures, and wrote the “Introduction,” and “Anatomy of the Motor System,” “Genetic Mutations and Risk Factors in ALS,” and “Intrinsic Factors Specific to MN Subpopulations” sections. JA conceived the article, wrote the “Conclusion” section, contributed text in numerous sections, and edited the manuscript throughout for content and style consistency.

## Conflict of Interest Statement

The authors declare that the research was conducted in the absence of any commercial or financial relationships that could be construed as a potential conflict of interest.

## Footnotes

1 http://alsod.iop.kcl.ac.uk/

2 https://www.jax.org/strain/002300
