# Supplementary material for: Motor Neuron Susceptibility in ALS/FTD
Source: Front Neurosci. 2019 Jun 27;13:532. doi: 10.3389/fnins.2019.00532 (PMC6610326; doi:10.3389/fnins.2019.00532)
Supplement: Supplementary file 1 [file Data_Sheet_1.docx]

| Gene | Protein | Locus | Frequency  in FALS (%) | Putative protein function | References |
| --- | --- | --- | --- | --- | --- |
| ***SOD1*** | Superoxide dismutase 1 | 21q22.11 | 12-23.5 | Scavenger enzyme, oxidative stress, UPS, autophagy | (Rosen et al., 1993; Andersen et al., 2003) |
| ***ANG*** | Angiogenin | 14q11.1 | 1.5 | Angiogenic factor | (Greenway et al., 2006; Wu et al., 2007; Bradshaw et al., 2017) |
| ***SQSTM1/p62*** | Sequestosome 1/p62 | 5q35 | 1.8 | Autophagy | (Fecto et al., 2011; Le Ber et al., 2013; Rubino et al., 2012) |
| ***DCTN1*** | Dynactin 1 | 2p13.1 | u | Axonal transport | (Münch et al., 2004; Liu et al., 2017; Puls et al., 2003; Münch et al., 2005) |
| ***VAPB*** | Vesicle-associated membrane protein (VAMP)-associated protein B | 20q13.33 | 0.6 | Vesicle trafficking, UPR | (Kabashi et al., 2013; Kuijpers et al., 2013; Nishimura et al., 2004) |
| ***VCP*** | Valosin-containing protein | 9p13.3 | 1-2.4 | Autophagy | (Johnson et al., 2010; DeJesus-Hernandez et al., 2011a) |
| ***TARDBP*** | TAR DNA binding protein-43 (TDP-43) | 1p36.22 | 5 | DNA/RNA metabolism | (Rutherford et al., 2008, 2008; Sreedharan et al., 2008; Kirby et al., 2010; Borroni et al., 2010) |
| ***FUS*** | Fused in sarcoma | 16p11.2 | 5 | DNA/RNA metabolism, stress granule function | (Belzil et al., 2009; Blair et al., 2009; Chiò et al., 2009; Kwiatkowski et al., 2009; Neumann et al., 2009; Vance et al., 2009) |
| ***ATXN2*** | Ataxin 2 | 12q24 | 5 | RNA translation, exocytosis | (Elden et al., 2010; Lin, 2011; Laffita-Mesa et al., 2013) |
| ***DAO*** | D-amino-acid oxidase | 13q33.2 | u | Oxidative deamination | (Millecamps et al., 2010; Mitchell et al., 2010) |
| ***OPTN*** | Optineurin | 10p13 | 2.6 | Autophagy | (Maruyama et al., 2010; Pottier et al., 2015) |
| ***C9orf72*** | Chromosome 9 open reading frame 72 | 9p21.2 | 30-50  (Europe, North America) | Endosomal trafficking, autophagy | (DeJesus-Hernandez et al., 2011b; Devenney et al., 2014; Farg et al., 2014; Majounie et al., 2012; Renton et al., 2011) |
| ***TAF15*** | TATA-binding associated factor | 17q12 | u | RNA metabolism | (Ticozzi et al., 2011) |
| ***UBQLN2*** | Ubiquilin 2 | Xp11.21 | 0.5-2.1 | Autophagy, UPS | (Deng et al., 2011; Kim et al., 2014, 2; Synofzik et al., 2012; Teyssou et al., 2017; Williams et al., 2012) |
| ***PFN1*** | Profilin-1 | 17p13 | 2.6 | Actin dynamics | (Wu et al., 2012; Dillen et al., 2013; Smith et al., 2014b) |
| ***hnRNPA1*** | Human heterogeneous nuclear ribonucleoprotein A1 | 12q13.13 | 0.5 | RNA metabolism | (Kim et al., 2013; Deshaies et al., 2018) |
| ***hnRNPA2B1*** | Human heterogeneous nuclear ribonucleoprotein A2B1 | 7p15.2 | u | RNA metabolism | (Kim et al., 2013) |
| ***CHCHD10*** | Coiled-coil-helix-coiled-coil-helix domain containing 10 | 22q11.23 | 3.6 | Mitochondrial function | (Bannwarth et al., 2014; Johnson et al., 2014a; Dols-Icardo et al., 2015; Zhang et al., 2015b; Perrone et al., 2017) |
| ***MATR3*** | Matrin 3 | 5q31.2 | 1.8 | RNA and DNA metabolism  mRNA nuclear export | (Feit et al., 1998; Johnson et al., 2014b; Müller et al., 2014; Boehringer et al., 2017) |
| ***TBK1*** | TANK-binding kinase 1 | 12q14.2 | 1-5.2 | Autophagy, inflammation | (Cirulli et al., 2015; Freischmidt et al., 2015; Pottier et al., 2015) |
| ***TUBA4A*** | Tubulin alpha-4A chain | 2q35 | 1.1 | Cytoskeleton | (Smith et al., 2014a; Perrone et al., 2017) |
| ***SCFD1*** | Sec1 family domain containing 1 | 14q12 | u | Vesicle transport | (van Rheenen et al., 2016) |
| ***MOBP*** | Myelin-Associated Oligodendrocyte Basic Protein | 3p22.1 | u | Compacting or stabilizing the myelin sheath | (van Rheenen et al., 2016; Yamamoto et al., 1994) |
| ***C21orf2*** | Chromosome 21 open reading frame 2 | 21q22.3 | 1.3-1.7 | Ciliogenesis, DNA damage repair | (Fang et al., 2015; Suga et al., 2016; van Rheenen et al., 2016) |
| ***CCNF*** | Cyclin F | 16p13.3 | 0.6-3.3 | UPS | (Williams et al., 2016) |
| ***NEK1*** | Never in mitosis gene A (NIMA)-related kinase 1 | 4q33 | u | Cell cycle control and cilia regulation, DNA damage repair | (Cirulli et al., 2015; Brenner et al., 2016; Higelin et al., 2016; Fang et al., 2015; Wheway et al., 2015; Polci et al., 2004) |
| ***NEFH*** | Neurofilament, heavy polypeptide 200kDa, heavy chain | 22q12.1 | 1 | Cytoskeleton | (Figlewicz et al., 1994) |

**Supplementary Table 1.** **Main genes implicated in familial and sporadic forms of ALS/FTD and their associated protein functions.**
